# Disability and schizophrenia: a systematic review of experienced psychosocial difficulties

**DOI:** 10.1186/1471-244X-12-193

**Published:** 2012-11-09

**Authors:** Piotr Świtaj, Marta Anczewska, Anna Chrostek, Carla Sabariego, Alarcos Cieza, Jerome Bickenbach, Somnath Chatterji

**Affiliations:** 1 I Department of Psychiatry, Institute of Psychiatry and Neurology, Sobieskiego 9, Warsaw, 02-957, Poland; 2 Research Unit for Biopsychosocial Health, Chair for Public Health and Health Care Research, Department of Medical Informatics, Biometry and Epidemiology (IBE), Ludwig-Maximilians-University, Munich, Germany; 3 Swiss Paraplegic Research, Nottwil, Switzerland; 4 Health Statistics and Informatics, WHO, Geneva, Switzerland

**Keywords:** Schizophrenia, Psychosocial difficulties, Disability, Functioning, ICF

## Abstract

**Background:**

Schizophrenia is a significantly disabling disease that affects all major areas of life. There is a lack of comprehensive synthesis of research findings on the full extent of psychosocial difficulties (PSDs) experienced by people living with schizophrenia. This paper provides a systematic review of the literature concerning PSDs and their associated factors in schizophrenia. PSDs were conceptualized in accordance with the International Classification of Functioning, Disability and Health (ICF) as disabilities, in particular impairments of mental functions, activity limitations and participation restrictions.

**Methods:**

An electronic search using MEDLINE and PsychINFO plus a manual search of the literature was performed for qualitative and longitudinal studies published in English between 2005 and 2010 that examined PSDs in persons with schizophrenia. The ICF was used as a conceptual framework.

**Results:**

A total of 104 papers were included. The most frequent PSDs addressed in the literature were not specific ones, directly linkable to the ICF categories of mental functions, activity limitations or participation restrictions, but broad areas of psychosocial functioning, such as psychopathological symptoms (53% of papers) or global disability and functioning (37%). Among mental functions, the most extensively studied were cognitive functions (27%) and emotional functions (27%). Within the domain of activities and participation, the most widely investigated were difficulties in relationships with others (31%) and employment (20%). Of the factors associated with the intensity or course of PSDs, the most commonly identified were treatment modalities (56%), psychopathological symptoms (26%), and socio-demographic variables (24%). Medication tended to improve the most relevant PSD, but at the same time was the only consistently reported determinant of onset of PSDs (emerging as unwanted side-effects).

**Conclusions:**

The present review illustrates the remarkably broad scope and diversity of psychosocial areas affected in schizophrenia and shows how these areas are interconnected and how they interact with contextual factors. The need for a shift in focus of schizophrenia research is suggested – from an excessive reliance on global measures of psychopathology and disability for defining outcomes to the creation of profiles of specific PSDs that have a more direct bearing on the disabling experience and real-world functioning of patients and can serve to guide interventions and monitoring over time.

## Background

Schizophrenia is a severe psychotic disorder characterized by a chronic and relapsing course with generally incomplete remissions, substantial functional decline, frequent psychiatric and medical co-morbidities and increased mortality [1]. Although several long-term follow-up studies have undermined the original view about its inevitably poor outcome and proved that varying degrees of recovery are possible [2], schizophrenia is still ranked among the top ten leading causes of disease-related disability in the world [3] and is consistently demonstrated to have a major negative impact on quality of life [4].

Given the magnitude and pervasiveness of the impairments associated with schizophrenia [1], only limited effectiveness of existing treatments [5], and the prevalence of stigma [6], people with schizophrenia commonly experience a wide and diverse array of psychosocial difficulties reaching far beyond the symptoms of the disease. Previous literature reviews analyzed various aspects of psychosocial disability in schizophrenia, such as psychopathological symptoms [7,8], or impairments of basic cognition [9], social cognition [10], emotional experience [11], social functioning [12], vocational functioning [13] and quality of life [14], but failed to systematically synthesize the data across the entire breadth of psychosocial problems experienced by people with this disease. Therefore, since the existing literature does not adequately reflect the overall experience and burden of living with schizophrenia, a review is needed to provide a comprehensive perspective on the totality of psychosocial difficulties associated with schizophrenia in order to allow for better intervention targeting and to suggest guidance for future research.

One of the main reasons the data on the entire range of schizophrenia-related psychosocial problems has not been satisfactorily summarized is the lack of consensus how to define psychosocial outcomes [15]. In the present review, we propose to define psychosocial difficulties (PSDs) according to the biopsychosocial approach found in the World Health Organization’s International Classification of Functioning, Disability and Health (ICF) [16]. The ICF provides a universal, common language for the description of health and health-related states, resulting from an interaction between the underlying health condition and contextual factors, namely environmental and personal factors. In other words, we do not regard PSDs to be direct consequences of schizophrenia, but mediated by the environment in which people with schizophrenia live. In accordance with the ICF framework, PSDs associated with schizophrenia can therefore be characterized as impairments of mental functions (such as emotional functions) and activity limitations and participation restrictions in such domains as work, family life and leisure activities. Environmental factors such as stigma, a supportive family as well as personal factors, such as confidence in one’s ability to overcome difficulties, can have a positive or negative impact on PSDs. Because PSDs are often analyzed by researchers in the context of several broader concepts, such as disability, functioning, quality of life, wellbeing or health status, we decided to include these notions in the analysis, even though we do not consider them to be specific PSDs. Also pain and sexual interest problems, which belong to the body functions component of the ICF but are not mental functions, were included due to their substantial psychological component and great importance in neuropsychiatric disorders.

Our review was performed within the scope of the EU funded project “Psychosocial fActors Relevant to brAin DISorders in Europe (PARADISE)” (http://paradiseproject.eu/), a project which aims to develop and test an innovative approach to collecting clinical data on PSDs that people with brain disorders experience in daily life. The key to this approach is the hypothesis that people with very distinct brain conditions – including dementia, depression, epilepsy, migraine, multiple sclerosis, Parkinson's disease, schizophrenia, stroke and substance use disorders – share common PSDs because, once again, these difficulties are not direct consequences of the health condition alone but are outcomes of the interaction between the health condition and contextual factors.

To provide a comprehensive overview of the most common PSDs in schizophrenia we reviewed qualitative and longitudinal studies targeting onset, intensity and course of PSDs experienced by adults with a diagnosis of schizophrenia. The specific objectives of the present systematic review were:

a) to acquire a comprehensive overview of the most common PSDs in schizophrenia;

b) to identify the most common factors associated with their onset, intensity and course; and

c) to identify gaps and weaknesses in the literature.

## Methods

### Search strategy

Electronic databases (MEDLINE and PsychINFO) were searched for studies published in English between January 2005 and May 2010 that examined psychosocial difficulties, as defined above, in persons with schizophrenia. Search terms were customized to each database by combining the terms schizophrenia and schizophren* with the following key words: psychosocial*, Quality of Life/, Personal Satisfaction/, exp Human Activities/ and exp Social Support/ disabilit*, homelessness, environmental factor*, exp Interpersonal Relations/, Quality of Life/, Personal Satisfaction/, exp Human Activities/, paternalism/, prejudice/, psychosocial deprivation/, social values/, exp Social Problems/, Social Adjustment/, social isolation/, stereotyping/, exp Social Environment/, exp emotions/, exp family/, exp socioeconomic factors/, exp life style/, exp Disability evaluation/, Communication Barriers/, Adaptation, Psychological/, Aggression/, Psychological stress/, (community not microbial community), or (sexual* or intimacy). The specific search strategies used in Medline and PsychINFO are presented in Additional file 1. Furthermore, reference lists of important systematic reviews targeting psychosocial difficulties in schizophrenia were screened for other relevant studies.

### Inclusion and exclusion criteria

With respect to the study population, papers were regarded as eligible for inclusion in the review if they focused on the perspective of adults with a diagnosis of schizophrenia. Studies performed on samples comprising patients with other psychotic disorders (e.g. schizotypal disorders, persistent delusional disorders, acute and transient psychotic disorders, induced psychotic disorders, or schizoaffective disorders) were excluded, unless the data concerning schizophrenia patients were analyzed and reported separately. Regarding study design, randomized controlled trials, clinical controlled trials, longitudinal observational studies, qualitative studies, case–control studies, economic evaluations, and study protocols were included. Excluded were primary prevention studies, phase I and II studies, ecologic studies, cross-sectional studies, systematic reviews, case reports/case series and psychometric studies. As far as publication type is concerned, only original research reports published as journal articles were considered for analysis, whereas book chapters, dissertations, guidelines, commentaries, letters to the editor, editorials, and conference reports were not eligible. Additional general exclusion criteria were as follows: absence of psychosocial factors analyzed as dependent variables, exclusive focus on caregiver’s burden, risk factors leading to schizophrenia, or environmental factors not considering the impact of those factors in persons with schizophrenia.

### Data extraction and analysis

All retrieved abstracts were screened for relevance by a trained reviewer. In order to enhance quality and reliability of this process, 20% of abstracts were randomly selected for a second check by another reviewer (blinded to the decision made by the other). Disagreements were resolved by consensus. Subsequently, full texts of papers rated as eligible were retrieved and analyzed in detail by one reviewer. 10% of full articles were double checked independently by two reviewers. The reviewers were psychiatrists with considerable experience in mental health research and practice (PŚ, ACh, MA).

Information from each study relevant to the research aims and which satisfied inclusion criteria was extracted. The extracted data referred to the study characteristics and to the PSDs reported. Information about study characteristics that was extracted was: the objective of the study, its design, intervention, if any, size and characteristics of the study population, outcome variables included, instruments used to assess them and research findings. The information extracted regarding PSDs was: the assessment method or instrument used, description of features of the PSD, such as onset (when the PSD began), intensity and nature of change over time (improvement, no change or worsening), and the factors related to PSDs. Variables were considered to be associated with PSDs if they showed statistically significant associations with the above mentioned features of PSDs in quantitative studies or were identified as such by the respondents in qualitative studies. Because in this study we were only interested in PSDs treated as dependent variables, we did not include here variables predicted by PSDs or which were analyzed in the papers as consequences of PSDs.

In the next step, the extracted PSDs were grouped into conceptually or thematically-related categories based on the ICF using the linking rules described by Cieza et al. [17]. The ICF standardized categories of PSDs were then grouped by similarity of content into overarching categories following guidelines for narrative synthesis described by Popay et al. [18]. A similar thematic categorization was performed also for the factors related to PSDs. In order to reduce the complexity of the data and the number of analyses needed, the factors associated with intensity and course of PSDs were analyzed jointly. A separate analysis was conducted for the factors associated with onset of PSDs. The importance of PSDs and associated factors was then assessed by calculating the number of studies in which they were identified. Only PSDs and associated variables which appeared in at least 5 papers were reported here. Subsequently, the types of relationships with associated variables were analyzed in detail for the 10 most frequent PSDs.

Finally, the quality of the studies was rated by investigators on a four-point scale (1-poor, 2-acceptable, 3-good, 4-excellent). The quality assessment was performed on the basis of the National Institute for Health and Clinical Excellence (NICE) guidelines [19]. Only papers of at least acceptable quality were retained for the final analysis.

## Results

### Determining relevant literature

**Figure 1:**
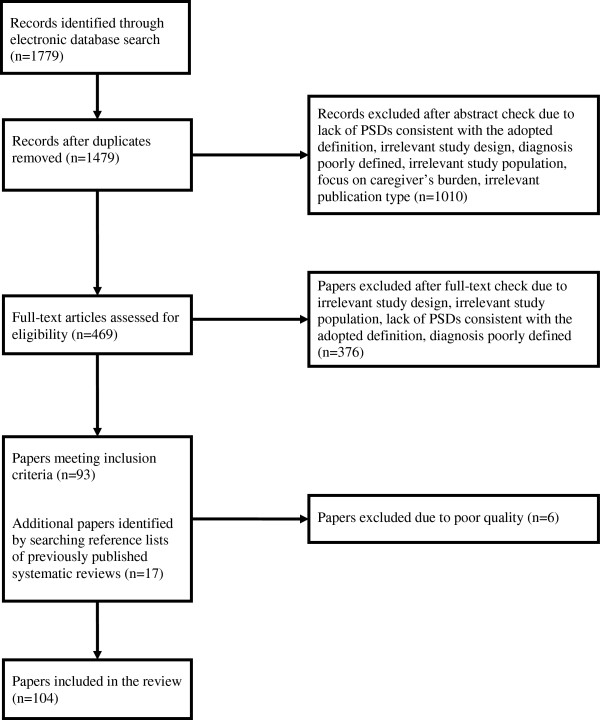
Flow diagram of paper selection process.

Study selection process is presented in Figure 1. A total of 104 papers formed part of the review (see Additional file 2 for the full list of the included papers).

### Characteristics of included studies

The overall quality of the included studies was rather high: 13 (12.5%) were rated as excellent, 72 (69.2%) were judged to be good, and 19 (18.3%) were assessed as acceptable. Seventeen (16.3%) studies were cross-national. Among the single country studies (n = 87, 83.7%), the most frequently represented countries were the USA (n = 18), Germany (n = 12), Spain (n = 9), and Israel (n = 7). The overwhelming majority of the studies (n = 94, 90.4%) were quantitative in nature, while only nine (8.7%) were qualitative, and one (1%) used a mixed-method design with both quantitative and qualitative data collected. The quantitative studies (including a quantitative part of the mixed-method study) were almost evenly split between interventional (n = 48) and observational studies (n = 47).

The study sample sizes varied from 4 to 9340 (median = 92) with a proportion of women ranging from 0% to 100% (median = 38.5%). Mean age of the participants was between 23.0 and 69.5 years. Only 25 (24%) studies reported mean time since illness onset for the sample which fell between 0.6 and 26.9 years. Additional file 3: Table S1 presents characteristics and a brief summary of findings of each study included in the review.

### Psychosocial difficulties (PSDs)

A total of 678 study outcomes related to PSDs were identified in the literature which were classified under 115 categories. The most relevant PSDs associated with schizophrenia are displayed in Table 1. ^a^

**Table 1:** **Frequency of psychosocial difficulties (PSDs) associated with schizophrenia identified in the literature**^**a**^

**PSD category (ICF code)**	**Papers in which a PSD category was assessed n (%)**
**Mental functions**	
**Cognitive functions**	**28 (26.9)**
in general (b1)	14 (13.5)
attention (b140)	11 (10.6)
memory (b144)	8 (7.7)
thought functions (b160)	8 (7.7)
insight (b1644)	6 (5.8)
executive functions (b164)	5 (4.8)
language (b167)	3 (2.9)
**Emotional functions**	**28 (26.9)**
depression (b1522)	16 (15.4)
anxiety (b152)	14 (13.5)
anhedonia (b1520)	5 (4.8)
hostility (b1522)	5 (4.8)
flat affect (b1522)	4 (3.8)
dysphoria (b1522)	2 (1.9)
feelings of stress (b152)	2 (1.9)
in general (b152)	2 (1.9)
mania (b152)	2 (1.9)
anger (b152)	1 (1.0)
emotional regulation (b1521)	1 (1.0)
emotional withdrawal (b152)	1 (1.0)
**Energy and drive**	**16 (15.4)**
motivation (b1301)	7 (6.7)
libido (b1308)	4 (3.8)
appetite (b1302)	3 (2.9)
fatigue (b1300)	3 (2.9)
vitality (b1300)	3 (2.9)
apathy (b130)	2 (1.9)
**Psychomotor functions (b147)**	**11 (10.6)**
**Sleep**	**10 (9.6)**
insomnia (b1340)	8 (7.7)
somnolence (b1340)	7 (6.7)
**Global psychosocial functions**	**9 (8.7)**
emotional perception (b122)	6 (5.8)
social cognition (b122)	5 (4.8)
**Sensory functions and pain**	
**Pain (b280)**	**11 (10.6)**
**Activities and participation**	
**Relationships with others**	**32 (30.8)**
in general (d7)	19 (18.3)
family relationships (d760)	7 (6.7)
aggressive behaviour (d7202)	5 (4.8)
inappropriate behaviour (d7202 + d7203)	5 (4.8)
intimate relationships (d770)	5 (4.8)
relationships with acquaintances (d7502)	3 (2.9)
relationships with health professionals (d7408)	2 (1.9)
behaviour at work (d740)	1 (1.0)
relationships with peers and colleagues (d7504)	1 (1.0)
**Employment**	**21 (20.2)**
in general (d850)	19 (18.3)
work efficiency (d850)	1 (1.0)
keeping employment (d8451)	1 (1.0)
obtaining employment (d845)	1 (1.0)
**Looking after ones’ health**	**12 (11.5)**
treatment adherence (d5702)	11 (10.6)
in general (d570)	1 (1.0)
**Participating in social activities (d9)**	**11 (10.6)**
**Self-care**	**11 (10.6)**
in general (d5)	10 (9.6)
personal hygiene and appearance (d598)	1 (1.0)
**Communication (d3)**	**7 (6.7)**
**Doing housework (d640)**	**6 (5.8)**
**Leisure activites (d920)**	**5 (4.8)**
**Global scores or global concepts closely related to PSDs**	
**Psychopathological symptoms**^**b**^	**55 (52.9)**
negative symptoms	39 (37.5)
positive symptoms	39 (37.5)
global intensity of symptoms	37 (35.6)
general psychopathology	18 (17.3)
**Disability and functioning**	**38 (36.5)**
global disability or global functioning	26 (25.0)
social functioning	18 (17.3)
**Quality of life and wellbeing**	**24 (23.1)**
quality of life	14 (13.5)
wellbeing	8 (7.7)
satisfaction in general	5 (4.8)
**Health status**	**12 (11.5)**
physical health	9 (8.7)
general health	6 (5.8)
psychological health	5 (4.8)
mental health	3 (2.9)
**Activities of daily living (ADL)**	**5 (4.8)**
**Other variables**	
**Skills**	**11 (10.6)**
social skills	8 (7.7)
general interpersonal skills	4 (3.8)
work skills	2 (1.9)
in general	2 (1.9)
communication skills	1 (1.0)
**Attitude towards treatment**	**5 (4.8)**
**Dependency**	**5 (4.8)**
in general	3 (2.9)
living independently	2 (1.9)
**Self-perception**	**5 (4.8)**
self-esteem	3 (2.9)
self-image	1 (1.0)
self-recognition	1 (1.0)

^a^ Only PSD categories which appeared in at least 5 papers were taken into account.

^b^ Under the general category of psychopathological symptoms were classified total scores of symptom rating scales, such as the PANSS or BPRS, or complex symptom clusters (positive symptoms, negative symptoms, or general psychopathology), which could not be linked to mental functions categories included in the ICF. More specific symptom dimensions derived from the PANSS or BPRS were placed under the respective categories of individual mental functions (e.g. emotional functions or energy and drive functions).

The most extensively studied were not concrete, specific PSDs, but global scores and global concepts closely related to PSDs, such as psychopathological symptoms, which were addressed in over a half (53%) of the included papers, or disability and functioning, measured in above a third (37%) of the papers. Other general aspects of life related to PSDs, which appeared in the literature with a significant frequency, were quality of life and wellbeing (23% of papers) and health status (12%). Within the ICF domain of mental functions, the most commonly assessed were cognitive functions (27%) and emotional functions (27%), followed by energy and drive (15%) and psychomotor functions (11%). Pain was also reported in 11% of papers. As far as the ICF component of activities and participation is concerned, the categories most widely investigated were relationships with others (31%), employment (20%), and, to a lesser extent, looking after one’s health (12%), participating in social activities (11%), and self-care (11%). Furthermore, several variables, which have not been comprehensively classified in the ICF so far, appeared in at least five papers, with various kinds of skills being the most frequently studied (11%).

Table 2 lists the outcome instruments used to assess PSDs in at least 5 papers. The most widely used were two symptom rating scales: PANSS (35% of papers) and BPRS (14%).

**Table 2:** **Outcome instruments most frequently used to assess PSDs**^**a**^

**Name of instrument**	**Papers in which an instrument was used n (%)**
Positive and Negative Syndrome Scale (PANSS) [20]	36 (34.6)
Brief Psychiatric Rating Scale (BPRS) [21]	15 (14.4)
Quality of Life Scale (QLS) [22]	7 (6.7)
Trail Making Test (TMT) [23]	7 (6.7)
Continuous Performance Test (CPT) [24]	6 (5.8)
Global Assessment of Functioning (GAF) [25]	6 (5.8)
Rey Auditory Verbal Learning Test (RAVLT) [26]	6 (5.8)
Scale for the Assessment of Negative Symptoms (SANS) [27]	6 (5.8)
Stroop Color and Word Test (SCWT) [28]	6 (5.8)
Subjective Well-Being Under Neuroleptic Treatment (SWN-K) [29]	6 (5.8)
World Health Organization Disability Assessment Schedule (WHODAS or WHODAS II) [30]	6 (5.8)
Clinical Global Impression-Schizophrenia scale (CGI-SCH) [31]	5 (4.8)
Montgomery-Åsberg Depression Scale (MADRS) [32]	5 (4.8)
Wechsler Adult Intelligence Scale (WAIS-R) [33]	5 (4.8)
Wechsler Memory Scale (WMS-R) [34]	5 (4.8)
Wisconsin Card Sorting Test (WCST) [35]	5 (4.8)

^a^ Only instruments which were used in at least 5 studies were included.

Instruments were only taken into account if they were used for assessing dependent variables.

### Variables associated with onset or course of PSDs

Variables associated with onset of PSDs were examined rarely and were reported only in a small proportion of papers (n = 17, 16.3%). Overall, seven categories of such variables were found. The only one consistently identified in the literature was medication, which was reported in 14 (13.5%) papers and found to contribute to the development of 14 various PSDs (as adverse events): most frequently pain (n = 11, 10.6%), anxiety (n = 8, 7.7%), insomnia (n = 8, 7.7%), somnolence (n = 7, 6.7%), increased appetite (n = 3, 2.9%), fatigue (n = 3, 2.9%), and loss of libido (n = 3, 2.9%). Other factors associated with onset of PSDs appeared in no more than two papers.

Factors related to the intensity or course of PSDs were studied much more frequently – 105 categories of associated variables were identified in 95 (91.3%) papers. The variables found in more than 5 papers are presented in Table 3 (included all variables which showed significant associations with any of the PSDs investigated in a given paper).

**Table 3:** **Frequency with which variables associated with intensity or course of PSDs were identified in the literature**^**a**^

**Associated variables**	**Papers in which a variable was identified n (%)**
**Patient treatment**	**58 (55.8)**
medication	32 (30.8)
psychosocial treatment	8 (7.7)
psychological therapy: cognitive therapy	7 (6.7)
alternative treatment	5 (4.8)
psychological therapy: cognitive behavioural therapy	4 (3.8)
community-based care	1 (1.0)
day care	1 (1.0)
**Psychopathological symptoms**	**27 (26.0)**
positive symptoms	16 (15.4)
negative symptoms	14 (13.5)
global intensity of symptoms	10 (9.6)
general psychopathology	3 (2.9)
**Demographics**	**25 (24.0)**
employment status	14 (13.5)
gender	12 (11.5)
marital status	8 (7.7)
educational level	7 (6.7)
age	6 (5.8)
accommodation type	6 (5.8)
country of residence	3 (2.9)
urban or rural residence	3 (2.9)
social economic status	2 (1.9)
disability benefits	1 (1.0)
legal status	1 (1.0)
**Disability and functioning**	**14 (13.5)**
global disability or global functioning	9 (8.7)
social functioning	7 (6.7)
**Emotional functions**	**13 (12.5)**
depression	10 (9.6)
feelings of stress	3 (2.9)
anxiety	2 (1.9)
dysphoria	1 (1.0)
flat affect	1 (1.0)
hostility	1 (1.0)
**Cognitive functions**	**12 (11.5)**
in general	6 (5.8)
insight	3 (2.9)
memory	3 (2.9)
attention	1 (1.0)
executive functions	1 (1.0)
intelligence	1 (1.0)
language	1 (1.0)
**Illness-related variables**	**10 (9.6)**
age at first hospitalization	3 (2.9)
duration of illness	3 (2.9)
stage of illness	3 (2.9)
age at first treatment	2 (1.9)
age at illness onset	2 (1.9)
course of illness	2 (1.9)
duration of untreated psychosis	2 (1.9)
subtype of schizophrenia	2 (1.9)
age at discharge from hospital	1 (1.0)
**Quality of life and wellbeing**	**9 (8.7)**
quality of life	5 (4.8)
wellbeing	5 (4.8)
**Social support**	**8 (7.7)**
in general	6 (5.8)
family support	1 (1.0)
friend support	1 (1.0)
**Comorbidities**	**7 (6.7)**
substance abuse	4 (3.8)
physical comorbidity	3 (2.9)
**Side-effects of medication**	**7 (6.7)**
**Treatment adherence**	**7 (6.7)**
**Relationships with others**	**6 (5.8)**
in general	3 (2.9)
family relationships	2 (1.9)
aggressive behaviour	1 (1.0)
relationships with health professionals	1 (1.0)
**Health services use**	**5 (4.8)**
duration of hospitalizations	3 (2.9)
number of hospitalizations	2 (1.9)
duration of treatment	1 (1.0)
number of health professional visits	1 (1.0)
**Psychomotor functions**	**5 (4.8)**
**Self-esteem**	**5 (4.8)**
**Support for caregivers**	**5 (4.8)**
education	5 (4.8)
in general	1 (1.0)
mutual support	1 (1.0)

a Only associated variables which were identified in at least 5 papers were taken into account.

The most common were various forms of treatment reported in over a half (56%) of the papers, followed by psychopathological symptoms (26%), socio-demographic variables (24%), global disability and functioning (13%), emotional functions (12.5%), and cognitive functions (12%).

Additional file 4: Table S2 shows how these variables were associated with the 10 most relevant PSD categories (negative findings are not included).

Most frequently associated with positive outcomes in psychosocial domains were various forms of treatment, particularly medication and psychosocial interventions. Psychopathological symptoms were most consistently related to negative outcomes.

## Discussion

In this paper we reviewed recent research on PSDs experienced by people with schizophrenia using a consistent conceptual framework for understanding the disability experience: that embodied in the internationally accepted standard of the ICF. Based on this conceptual framework, we defined PSDs as impairments of mental functions (including also pain and sexual interest functions), activity limitations and participation restrictions. So our approach was broader and more comprehensive than adopted by those authors who tend to exclude disturbances of mental functions (e.g. psychopathological symptoms or cognitive deficits) from the definition of psychosocial functioning [15,36]. It should be emphasized as well, that unlike a large part of earlier reviews targeting psychosocial problems of people with schizophrenia, we excluded studies conducted on diagnostically heterogeneous samples, comprising people with schizoaffective or other psychotic disorders. The exclusive focus on people diagnosed with schizophrenia is a strength of this paper, given the unclear nosological status of schizoaffective disorder and a low reliability, longitudinal stability and clinical utility of this diagnosis, which is strongly recommended by prominent researchers in the field to be deleted from future revisions of the classifications of mental disorders [1].

The analysis of the included papers resulted in identifying more than a hundred PSD categories, which clearly confirms that psychosocial problems encountered by people with schizophrenia in their daily lives are very diverse. The most frequently addressed PSDs were related to the areas of psychopathology, overall disability and functioning, relationships with others, cognitive functions, emotional functions, quality of life and wellbeing, employment, and energy and drive. This pattern of findings well reflects the core features of schizophrenia as a disabling disease manifesting itself by an admixture of positive, negative, cognitive, mood and motor symptoms, variable degrees of functional, social and occupational impairments, and marked worsening of both objective and subjective indicators of quality of life [1].

Our analysis also revealed a wide variety of over a hundred categories of factors associated with the intensity or course of PSDs. Of those, by far the most commonly reported (in over 50% of the papers) were treatment modalities, with medication being the most frequent, followed by psychological and psychosocial therapies. Therapeutic interventions generally had positive influence on PSDs, but only medication and to a lesser extent psychosocial treatment were consistently reported to positively affect a majority of the most relevant PSDs, whereas the effects of other forms of therapy were more selective. It should be borne in mind, though, that the beneficial effects of medication came at a price, because it was also found to be a determinant of onset of quite a few PSDs.

The second most frequent group of factors associated with PSDs were factors that are psychosocial problems themselves, e.g. psychopathological symptoms, global disability and functioning, emotional problems, or cognitive deficits. It comes as no surprise that psychiatric symptoms in particular were found to be associated with negative outcomes in most of the main psychosocial domains as they are a primary source of suffering and life problems of schizophrenia patients. In more general terms, our findings illustrate how various areas of psychosocial functioning in schizophrenia are closely interrelated and therefore cannot be targeted and effectively ameliorated in isolation.

Socio-demographic variables emerged as the third particularly relevant group of variables related to the intensity or course of PSDs. However, in this case the pattern of relationships was much less clear, with working status being the most consistently found to be associated with positive outcomes, and male gender most often being related to negative outcomes. An earlier review on schizophrenia and employment by Marwaha & Johnson [13] showed the correlation of being employed with better social functioning, quality of life and self-esteem, and with lower symptom levels, but at the same time stressed the scarcity of evidence for the causal relationships. Male gender, in turn, is a well known predictor of poor outcome of schizophrenia [3]. Overall, our review suggests that socio-demographic characteristics play an important role in shaping the daily experience of persons with schizophrenia, but their interactions with psychosocial outcomes are complex and difficult to synthesize. In the framework of the ICF, socio-demographic characteristics belong to the domain of contextual personal factors, which until now has only preliminarily been outlined [16]. A systematic exploration of the conceptualization and operationalization of personal factors in the ICF would help in studying them more rigorously and at the same time provide a more complete understanding of the experience of disability in persons with schizophrenia.

The present review points to several gaps and weaknesses in the literature. First of all, it should be noted that the most widely studied were not specific PSDs in a strict sense, directly linkable to the ICF categories of mental functions, activity limitations or participation restrictions, but rather broad areas of psychosocial functioning, such as global severity of psychopathological symptoms or complex symptom clusters (e.g. positive or negative symptoms), overall disability and functioning. Other general aspects of life that, with significant frequency, were related to psychosocial functioning in schizophrenia included quality of life, wellbeing and various dimensions of health status. Although all these broad constructs may be useful as outcome measures in evaluating the effectiveness of therapeutic interventions, they provide only limited information on psychosocial problems actually experienced by people with schizophrenia in real life. It is desirable that future research should place more emphasis on specific areas of psychosocial disability in schizophrenia which could be more direct targets for interventions and policy changes.

Further, the relevant psychosocial areas that are underrepresented in the literature need to be mentioned. In the light of the widely accepted view that the stigma associated with schizophrenia is particularly high and constitutes one of the main obstacles to social inclusion and to better quality of life of people who have the illness [6], it is surprising that perception and experience of stigma was a rarely investigated PSD in the reviewed studies (below our threshold of 5 papers). Thus, although recent decades witnessed a marked increase in published research on psychiatric stigma [37], this trend was not reflected in the present review. One explanation may be that most studies examining stigma and discrimination from the perspective of people with mental illness use cross-sectional designs and samples not meeting our inclusion criteria regarding diagnosis [38]. However, the fact that in the included papers experience of stigma was reported very infrequently not only as a PSD, but also as a determinant of other PSDs (again, below the set threshold of 5 articles) may indicate that it is indeed still insufficiently taken into account in schizophrenia research.

Another important schizophrenia-related PSD category that seems to be understudied is that of temperament and personality functions, which were also addressed in fewer than five articles. This is in contrast with the results of two recent reviews focusing on the disability of people with affective disorders (using the ICF as a reference), in which temperament and personality functions were found to be among the most relevant psychosocial factors [39,40]. It is even more surprising that in the present review personality dimensions were rarely (< 5 papers) identified as determinants of PSDs, because there exists evidence for their substantial impact on symptoms and various aspects of functioning in people with psychotic disorders [41,42]. It appears, then, that schizophrenia research concerning psychosocial functioning is focused first and foremost on psychopathology and functional outcomes, while personal dispositions of people with schizophrenia is a largely neglected and underestimated area.

We found that the most frequently used instruments assessing PSDs were two symptom rating scales: PANSS and BPRS. Although these scales have dominated the field of schizophrenia research, their value as outcome measures is rather limited and is increasingly questioned. It is argued that simply rating symptoms, without careful evaluation of cognition, personal and social functioning, is of little practical use [43]. Our review suggests that schizophrenia research suffers from overreliance on clinician-rated symptoms in the determination of outcome. In the analyzed studies, all too often the outcome measurement was based primarily on the global rating of the severity of symptoms by means of the standardized scales, with little attention being paid to a deeper investigation of specific aspects of psychopathology and to the assessment of other domains of psychosocial functioning. It is also problematic that patient’s perspective on the psychic phenomena, which are a crucial aspect of their disease was largely overlooked. The self-assessment symptom measures do not have a strong tradition in schizophrenia research because of the objections to their validity, which contrasts with their popularity in other mental disorders such as depression and anxiety [44]. It seems, however, that, despite their inherent limitations, their wider use might help in capturing the subjective experience of the patients and thus in narrowing potential gap between clinicians’ and patients’ perspectives. Besides assessments made by trained professionals with regard to PSDs associated with schizophrenia, future research needs to also focus on individual importance attached to these PSDs such that interventions can be appropriately prioritized.

The frequency of use of instruments assessing other relevant psychosocial areas, such as cognition, depression, social functioning, disability or quality of life demonstrates that no measure played a similarly prominent role in the literature as the PANSS and BPRS in the field of psychopathology assessment. This confirms the conclusions of other researchers [36] that, so far, there is no universally acknowledged gold standards in these domains, which highlights the need for the development of new outcome measures or better validation of the existing ones.

Finally, it is worth noticing that in contrast to variables associated with the intensity or course of PSDs, factors related to the onset of PSDs were rarely reported in the literature (with the notable exception of medication and its side-effects). One main reason is probably the practical difficulty in designing studies that would be able to capture the onset of PSDs and factors contributing to it. A more extensive qualitative research on PSDs in schizophrenia should be undertaken in order to hear directly from the patients how their life problems started and how they perceived their causes.

Our review should be interpreted in the light of several limitations. First, we did not analyze papers published in languages other than English. Thus, a possibility exists that important findings from non-English speaking countries were not taken into account. Second, only a small proportion of the included studies came from low- and middle-income countries. This restricts the generalizability of the results, given that many psychosocial problems in schizophrenia may be to a large extent dependent on economic, political and cultural factors. Third, we only included longitudinal studies because these provide higher level of evidence, especially in relation to the link between PSDs and associated variables. However, it is necessary to take into account when interpreting the results of this review that cross-sectional studies make up the lion’s share of schizophrenia research. Fourth, we only analyzed studies which conceptualized PSDs as dependent variables and cannot make inferences about which and how PSDs are used as independent variables. Finally, our analysis of the relationships between the PSDs and other variables does not allow for inferring causality. Because of the huge amount and heterogeneity of analyzed variables, as well as the great variability in study designs (including both quantitative and qualitative research methods), types of statistical analyses employed and the quality of reporting the results, we decided to synthesize the data only at the most basic level of associations.

## Conclusions

The present review illustrates the remarkably broad scope and diversity of psychosocial areas affected in schizophrenia. It shows how these areas are interconnected and how they interact with contextual factors, both environmental (e.g. treatments received) and personal ones (e.g. socio-demographic characteristics). Therefore, it stresses the need for a comprehensive approach to schizophrenia-related PSDs in research and clinical practice, since they can not be properly understood and effectively improved in isolation and without careful attention to the specific context in which they appear.

Our findings point to the necessity of a shift in focus of schizophrenia research from an excessive reliance on global measures of psychopathology and disability in the determination of outcome to a fuller consideration of specific PSDs that have a more direct bearing on the real-world functioning of the schizophrenia sufferers. Researchers should also pay more attention to the investigation of perceptions and experiences of stigma, as well as temperament and personality functions of people with schizophrenia. A deeper exploration of these understudied areas not only will shed more light on the extent of psychosocial problems faced by the patients, but will also be helpful in uncovering their personal strengths that make them less vulnerable to disability and social exclusion. As well, it would be advisable to put more emphasis on the analysis of the determinants of onset of PSDs.

Although up until now the application of the ICF in psychiatry was rather limited [45], this review demonstrates its usefulness in conceptualizing PSDs experienced by schizophrenia patients. The further development of the ICF (including an elaboration of personal factors) and its wider implementation appears to be a promising means to a more systematic and comprehensive investigation of various dimensions of psychosocial disabilities in schizophrenia along with their determinants. The ICF can also serve as a basis for creating instruments comprehensively assessing psychosocial outcomes in schizophrenia that could be of use in both research and clinical praxis. Understanding profiles of patients of schizophrenia at different stages of the illness and over time would enable more effective matching of interventions, such as skills training, and monitoring their impact over time in terms of what really matters to patients and caregivers.

## Endnotes

^a^For the sake of simplicity, PSD categories were formulated in neutral language (e.g. “cognitive functions” instead of “impairments in cognitive functions”).

## Abbreviations

PSDs: Psychosocial difficulties; ICF: International Classification of Functioning, Disability and Health.

## Competing interests

The authors declare that they have no competing interests.

## Authors’ contributions

PŚ was the main reviewer, analyzed and interpreted the data, and drafted the manuscript. ACh was the second reviewer and took part in the data analysis. MA was involved in reviewing the abstracts and contributed to the interpretation of the data. CS conducted the literature searches and contributed to the analysis and interpretation of the data. AC, JB and SC conceived the study aims and design. All authors reviewed the draft manuscript and read and approved the final manuscript.

## Pre-publication history

The pre-publication history for this paper can be accessed here:

http://www.biomedcentral.com/1471-244X/12/193/prepub

## Supplementary Material

Additional file 1 Search strategies used in Medline and PsychInfo. Click here for file

Additional file 2 Papers included in the systematic review. Click here for file

Additional file 3 Table S1 Characteristics and main findings of included studies. Click here for file

Additional file 4 Table S2 Variables associated with the intensity or course of the 10 most frequently studied PSD categories. Click here for file
