# Supplementary material for: Disability and schizophrenia: a systematic review of experienced psychosocial difficulties
Source: BMC Psychiatry. 2012 Nov 9;12:193. doi: 10.1186/1471-244X-12-193 (PMC3539983; doi:10.1186/1471-244X-12-193)
Supplement: Additional file 1 — Search strategies used in Medline and PsychInfo. [file 1471-244X-12-193-S1.pdf]

Additional File 1

Search strategies used in Medline and PsychInfo

Final Search Medline

1. exp SCHIZOPHRENIA/
2. schizophren*.ti,ab.
3. 1 and 2
4. exp Animals/ not Humans/
5. (mouse or mice or rat or rats or rabbit or rabbits or guinea?pig* or animal model*).ti,ab.
6. clinical trial, phase i/ or clinical trial, phase ii/ or case reports/ or exp in vitro/
7. exp Cross-Sectional Studies/
8. exp longitudinal studies/ or exp follow-up studies/ or exp prospective studies/
9. 7 not 8
10. 4 or 5 or 6 or 9
11. 3 not 10
12. limit 11 to (abstracts and english language and humans and yr="2005 -Current")
13. limit 11 to ("all infant (birth to 23 months)" or "all child (0 to 18 years)")
14. 12 not 13
15. psychosocial*.mp. or Quality of Life/ or Personal Satisfaction/ or exp Human Activities/ or Social Support/
16. (disabilit* not "down syndrome").ti,ab. or exp Social Problems/ or Social Adjustment/
17. exp Interpersonal Relations/ or prejudice/ or social isolation/ or exp emotions/ or exp life style/ or (sexual* or intimacy).mp.
18. or/15-17
19. 14 and 18
20. schizophrenia/ge or schizophrenia/me or schizophrenia/pp or emotions/ph
21. 19 not 20
22. exp Review/
23. exp Review Literature as Topic/
24. ((systemat* or critical) adj (review* or search*)).ti.
25. ((systematic or critical) adj (literature or narrative or qualitative or quantitative or evidence or evidence based or Cochrane) adj (review* or search*)).ti.
26. evidence based review.ti.
27. or/22-26
28. 21 not 27

Final Search PsychInfo

Limiters - Publication Year from: 2005-2010; English; Language: English; Population Group: Human

1. MJ "Schizophrenia"

2. TI schizophren* or AB schizophren*

3. S1 and S2

4. PO (animal not human)

5. TI (mouse or mice or rat or rats or rabbit or rabbits or animal model*) or AB (mouse or mice or rat or rats or rabbit or rabbits or animal model*)

6. MM "Clinical Trials" or MM "Case Report"

7. TI Cross-Sectional* or AB Cross-Sectional* or DE "Surveys"

8. DE "Followup Studies" or DE "Longitudinal Studies" OR DE "Prospective Studies"

9. S7 not S8

10. S4 or S5 or S6 or S9

11. S3 not S10

12. TX psychosocial* or MJ Quality of Life/ or MJ Life Satisfaction/ or MJ "Unemployment" or MJ "Employability" or MJ "Reemployment" or MJ "Retirement" or MJ "Traveling" or MJ "Leisure Time" or MJ "Exercise" or MJ "Physical Activity" or MJ "Physical Fitness" or MJ "Driving Behavior" or MJ "Aggressive Driving Behavior" or MJ "Driving Under the Influence" or MJ Activities of Daily Living/ or MJ Social Support/

13. TX disabilit*

14. MM "Social Discrimination" OR MM "Age Discrimination" OR MM "Disability Discrimination" OR MM "Employment Discrimination" OR MM "Race and Ethnic Discrimination" OR MM "Sex Discrimination" or MM "Discrimination" or MM "Social Issues" or MM "Disability Discrimination" or MM "Employment Discrimination" or MM "Social Integration" OR MM "School Integration"

15. DE "Interpersonal Relationships" or MM "Interpersonal Relationships" OR MM "Family Relations" OR MM "Friendship" OR MM "Kinship" OR MM "Marital Relations"

16. DE "Prejudice" or MM "Prejudice" or MM "Social Isolation"

17. MM "Emotional States" OR MM "Affection" OR MM "Alienation" OR MM "Ambivalence" OR MM "Anger" OR MM "Anxiety" OR MM "Apathy" OR MM "Aversion" OR MM "Boredom" OR MM "Depression (Emotion)" OR MM "Disappointment" OR MM "Disgust" OR MM "Dissatisfaction" OR MM "Distress" OR MM "Doubt" OR MM "Embarrassment" OR MM "Emotional Trauma" OR MM "Enthusiasm" OR MM "Euphoria" OR MM "Fear" OR MM "Frustration" OR MM "Gratitude" OR MM "Grief" OR MM "Guilt" OR MM "Happiness" OR MM "Helplessness" OR MM "Homesickness" OR MM "Hope" OR MM "Hopelessness" OR MM "Jealousy" OR MM "Loneliness" OR MM "Love" OR MM "Mania" OR MM "Mental Confusion" OR MM "Optimism" OR MM "Pessimism" OR MM "Pleasure" OR MM "Pride" OR MM "Regret" OR MM "Restlessness" OR MM "Sadness" OR MM "Shame" OR MM "Suffering" OR MM "Suspicion" OR MM "Sympathy" or MM "Emotions" OR MM "Desire" OR MM "Emotional States" or MM "Affection" or MM "Ambivalence" or MM "Anger" or MM "Anxiety" or DE "Apathy" or MM

"Aversion" or MM "Boredom" or MM "Depression (Emotion)" or DE "Disappointment" or MM "Euphoria" or DE "Fear" or MM "Happiness" or DE "Helplessness"

18. TX sexual* or TX intimacy

19. S12 or S13 or S14 or S15 or S16 or S17 or S18

20. S11 and S19
