# Supplementary material for: Disability and schizophrenia: a systematic review of experienced psychosocial difficulties
Source: BMC Psychiatry. 2012 Nov 9;12:193. doi: 10.1186/1471-244X-12-193 (PMC3539983; doi:10.1186/1471-244X-12-193)
Supplement: Additional file 2 — Papers included in the systematic review. [file 1471-244X-12-193-S2.pdf]

Additional File 2

Papers included in the systematic review

1. Addington J, Saeedi H, Addington D: **Facial affect recognition: a mediator between cognitive and social functioning in psychosis?** *Schizophr Res* 2006, **85:**142-150.
2. Addington J, Saeedi H, Addington D: **Influence of social perception and social knowledge on cognitive and social functioning in early psychosis.** *Br J Psychiatry* 2006, **189:**373-378.
3. Alonso J, Croudace T, Brown J, Gasquet I, Knapp MRJ, Suárez D, Novick D: **Health-related quality of life (HRQL) and continuous antipsychotic treatment: 3-year results from the Schizophrenia Health Outcomes (SOHO) study.** *Value Health* 2009, **12:**536-543.
4. Alptekin K, Erkoç S, Göğüş AK, Kültür S, Mete L, Uçok A, Yazici KM: **Disability in schizophrenia: clinical correlates and prediction over 1-year follow-up.** *Psychiatry Res* 2005, **135:**103-111.
5. Álvarez E, Ciudad A, Olivares JM, Bousoño M, Gómez JC: **A randomized, 1-year follow-up study of olanzapine and risperidone in the treatment of negative symptoms in outpatients with schizophrenia.** *J Clin Psychopharmacol* 2006, **26:**238-249.
6. Arango C, Bombín I, González-Salvador T, García-Cabeza I, Bobes J: **Randomised clinical trial comparing oral versus depot formulations of zuclopenthixol in patients with schizophrenia and previous violence.** *Eur Psychiatry* 2006, **21:**34-40.
7. Beebe LH, Smith K, Crye C, Addonizio C, Strunk DJ, Martin W, Poche J: **Telenursing intervention increases psychiatric medication adherence in schizophrenia outpatients.** *J Am Psychiatr Nurses Assoc* 2008, **14:**217-224.

8. Beebe LH, Tian L, Morris N, Goodwin A, Allen SS, Kuldau J: **Effects of exercise on mental and physical health parameters of persons with schizophrenia.** *Issues Ment Health Nurs* 2005, **26:**661-676.
9. Bejerholm U, Eklund M: **Engagement in occupations among men and women with schizophrenia.** *Occup Ther Int* 2006, **13:**100-121.
10. Bitter I, Basson BR, Dossenbach MR: **Antipsychotic treatment and sexual functioning in first-time neuroleptic-treated schizophrenic patients.** *Int Clin Psychopharmacol* 2005, **20:**19-21.
11. Borras L, Mohr S, Boucherie M, Dupont-Willemin S, Ferrero F, Huguelet P: **Patients with schizophrenia and their finances: how they spend their money**. *Soc Psychiatry Psychiatr Epidemiol* 2007, **42:**977-983.
12. Breier A, Berg PH, Thakore JH, Naber D, Gattaz WF, Cavazzoni P, Walker DJ, Roychowdhury SM, Kane JM: **Olanzapine versus ziprasidone: results of a 28-week double-blind study in patients with schizophrenia.** *Am J Psychiatry* 2005, **162:**1879–1887.
13. Buizza C, Schulze B, Bertocchi E, Rossi G, Ghilardi A, Pioli R: **The stigma of schizophrenia from patients' and relatives' view: a pilot study in an Italian rehabilitation residential care unit.** *Clin Pract Epidemiol Ment Health* 2007, **3:**23.
14. Cavallaro R, Anselmetti S, Poletti S, Bechi M, Ermoli E, Cocchi F, Stratta P, Vita A, Rossi A, Smeraldi E: **Computer-aided neurocognitive remediation as an enhancing strategy for schizophrenia rehabilitation.** *Psychiatry Res* 2009, **169:**191-196.
15. Ceccato E, Caneva P, Lamonaca D: **Music therapy and cognitive rehabilitation in schizophrenic patients: a controlled study.** *Nord J Music Ther* 2006, **15:**111-120.

16. Chang JS, Yi JS, Ahn YM, Kim JH, Kim YS: **Stabilization of the internal structure of persistent auditory verbal hallucinations in schizophrenia.** *Aust N Z J Psychiatry* 2009, **43:**244-251.
17. Chien WT, Chan SWC, Thompson DR: **Effects of a mutual support group for families of Chinese people with schizophrenia: 18-month follow-up.** *Br J Psychiatry* 2006, **189:**41-49.
18. Chien WT, Thompson DR, Norman I: **Evaluation of a peer-led mutual support group for Chinese families of people with schizophrenia.** *Am J Community Psychol* 2008, **42:**122-134.
19. Chien WT, Wong KF: **A family psychoeducation group program for Chinese people with schizophrenia in Hong Kong.** *Psychiatr Serv* 2007, **58:**100-106.
20. Chrzanowski WK, Marcus RN, Torbeyns A, Nyilas M, McQuade RD: **Effectiveness of long-term aripiprazole therapy in patients with acutely relapsing or chronic, stable schizophrenia: a 52-week, open-label comparison with olanzapine.** *Psychopharmacology* 2006, **189:**259-266.
21. Conley RR, Kelly DL, Nelson MW, Richardson CM, Feldman S, Benham R, Steiner P, Yu Y, Khan I, McMullen R, Gale E, Mackowick M, Love RC: **Risperidone, quetiapine, and fluphenazine in the treatment of patients with therapy-refractory schizophrenia.** *Clin Neuropharmacol* 2005, **28:**163–168.
22. Dossenbach M, Dyachkova Y, Pirildar S, Anders M, Khalil A, Araszkiewicz A, Shakhnovich T, Akram A, Pecenak J, McBride M, Treuer T: **Effects of atypical and typical antipsychotic treatments on sexual function in patients with schizophrenia: 12-month results from the Intercontinental Schizophrenia Outpatient Health Outcomes (IC-SOHO) study.** *Eur Psychiatry* 2006, **21:**251-258.

23. Fisher M, Holland C, Merzenich MM, Vinogradov S: **Using neuroplasticity-based auditory training to improve verbal memory in schizophrenia.** *Am J Psychiatry* 2009, **166:**805-811.

24. Fuentes I, García S, Ruiz JC, Soler MJ, Roder V: **Social perception training in schizophrenia: a pilot study.** *Intern Jour Psych Psychol Ther* 2007, **7:**1-12.

25. Gelkopf M, Gonen B, Kurs R, Melamed Y, Bleich A: **The effect of humorous movies on inpatients with chronic schizophrenia.** *J Nerv Ment Dis* 2006, **194:**880-883.

26. Gil Sanz D, Diego Lorenzo M, Bengochea Seco R, Arrieta Rodríguez M, Lastra Martínez I, Sánchez Calleja R, Álvarez Soltero A: **Efficacy of a social cognition training program for schizophrenic patients: a pilot study.** *Span J Psychol* 2009, **12:**184-191.

27. González-Torres MA, Oraa R, Arístegui M, Fernández-Rivas A, Guimon J: **Stigma and discrimination towards people with schizophrenia and their family members. A qualitative study with focus groups.** *Soc Psychiatry Psychiatr Epidemiol* 2007, **42:**14-23.

28. Górna K, Jaracz K, Rybakowski F, Rybakowski J: **Determinants of objective and subjective quality of life in first-time-admission schizophrenic patients in Poland: a longitudinal study.** *Qual Life Res* 2008, **17:**237-247.

29. Gray R, Leese M, Bindman J, Becker T, Burti L, David A, Gournay K, Kikkert M, Koeter M, Puschner B, Schene A, Thornicroft G, Tansella M: **Adherence therapy for people with schizophrenia. European multicentre randomised controlled trial.** *Br J Psychiatry* 2006, **189:**508-514.

30. Grover S, Avasthi A, Chakrabarti S, Bhansali A, Kulhara P: **Cost of care of schizophrenia: a study of Indian out-patient attenders.** *Acta Psychiatr Scand* 2005, **112:**54-63.

31. Gur RE, Kohler CG, Ragland JD, Siegel SJ, Lesko K, Bilker WB, Gur RC: **Flat affect in schizophrenia: relation to emotion processing and neurocognitive measures.** *Schizophr Bull* 2006, **32:**279-287.

32. Harrow M, Grossman LS, Jobe TH, Herbener ES: **Do patients with schizophrenia ever show periods of recovery? A 15-year multi-follow-up study.** *Schizophr Bull* 2005, **31:**723-734.

33. Heider D, Angermeyer MC, Winkler I, Schomerus G, Bebbington PE, Brugha T, Azorin JM, Toumi M: **A prospective study of quality of life in schizophrenia in three European countries.** *Schizophr Res* 2007, **93:**194-202.

34. Herbener ES, Hill SK, Marvin RW, Sweeney JA: **Effects of antipsychotic treatment on emotion perception deficits in first-episode schizophrenia.** *Am J Psychiatry* 2005, **162:**1746-1748.

35. Hill A, Mayes R, McConnell D: **Transition to independent accommodation for adults with schizophrenia.** *Psychiatr Rehabil J* 2010, **33:**228-231.

36. Honkonen T, Stengård E, Virtanen M, Salokangas RKR: **Employment predictors for discharged schizophrenia patients.** *Soc Psychiatry Psychiatr Epidemiol* 2007, **42:**372-380.

37. Horan WP, Ventura J, Mintz J, Kopelowicz A, Wirshing D, Christian-Herman J, Foy D, Liberman RP: **Stress and coping responses to a natural disaster in people with schizophrenia.** *Psychiatry Res* 2007, **151:**77-86.

38. Inadomi H, Tanaka G, Watanabe S, Nagatomi Y, Mitarai K, Ohsawa R, Kusumeki K, Shimatani T, Hatanaka K, Hiroike T, Teramoto K, Utsunomiya H, Etoh R, Ohta Y:

Efficacy of 3-year psychiatric daycare treatment in patients with schizophrenia. *Psychiatry Clin Neurosci* 2005, **59:**246-252.

39. Jockers-Scherübl MC, Bauer A, Godemann F, Reischies FM, Selig F, Schlattmann P: **Negative symptoms of schizophrenia are improved by the addition of paroxetine to neuroleptics: a double-blind placebo-controlled study.** *Int Clin Psychopharmacol* 2005, **20:**27–31.
40. Kallert TW, Leisse M, Winiecki P: **Comparing the effectiveness of different types of supported housing for patients with chronic schizophrenia.** *J Public Health* 2007, **15:**29-42.
41. Kane JM, Khanna S, Rajadhyaksha S, Giller E: **Efficacy and tolerability of ziprasidone in patients with treatment-resistant schizophrenia.** *Int Clin Psychopharmacol* 2006, **21:**21–28.
42. Kane JM, Meltzer HY, Carson WH, McQuade RD, Marcus RN, Sanchez R, for the Aripiprazole Study Group: **Aripiprazole for treatment-resistant schizophrenia: results of a multicenter, randomized, double-blind, comparison study versus perphenazine.** *J Clin Psychiatry* 2007, **68:**213–223.
43. Karow A, Czekalla J, Dittmann RW, Schacht A, Wagner T, Lambert M, Schimmelmann BG, Naber D: **Association of subjective well-being, symptoms, and side effects with compliance after 12 months of treatment in schizophrenia.** *J Clin Psychiatry* 2007, **68:**75-80.
44. Keefe RSE, Bilder RM, Davis SM, Harvey PD, Palmer BW, Gold JM, Meltzer HY, Green MF, Capuano G, Stroup S, McEvoy JP, Swartz MS, Rosenheck RA, Perkins DO, Davis CE, Hsiao JK, Lieberman JA, for the CATIE Investigators and the Neurocognitive Working Group: **Neurocognitive effects of antipsychotic**

medications in patients with chronic schizophrenia in the CATIE Trial. *Arch Gen Psychiatry* 2007, **64:**633-647.

45. Kolotkin RL, Corey-Lisle PK, Crosby RD, Kan HJ, McQuade RD: **Changes in weight and weight-related quality of life in a multicentre, randomized trial of aripiprazole versus standard of care.** *Eur Psychiatry* 2008, **23:**561-566.
46. Konarzewska B, Wołczyński S, Szulc A, Galińska B, Popławska R, Waszkiewicz N: **Effect of risperidone and olanzapine on reproductive hormones, psychopathology and sexual functioning in male patients with schizophrenia.** *Psychoneuroendocrinology* 2009, **34:**129-139.
47. Kulhara P, Chakrabarti S, Avasthi A, Sharma A, Sharma S: **Psychoeducational intervention for caregivers of Indian patients with schizophrenia: a randomised-controlled trial.** *Acta Psychiatr Scand* 2009, **119:**472-483.
48. Lambert M, Naber D, Schacht A, Wagner T, Hundemer HP, Karow A, Huber CG, Suarez D, Haro JM, Novick D, Dittmann RW, Schimmelmann BG: **Rates and predictors of remission and recovery during 3 years in 392 never-treated patients with schizophrenia.** *Acta Psychiatr Scand* 2008, **118:**220-229.
49. Lambert M, Schimmelmann BG, Naber D, Schacht A, Karow A, Wagner T, Czekalla J: **Prediction of remission as a combination of symptomatic and functional remission and adequate subjective well-being in 2960 patients with schizophrenia.** *J Clin Psychiatry* 2006, **67:**1690-1697.
50. Lambert M, Schimmelmann BG, Schacht A, Karow A, Wagner T, Wehmeier PM, Huber CG, Hundemer HP, Dittmann RW, Naber D: **Long-term patterns of subjective wellbeing in schizophrenia: cluster, predictors of cluster affiliation, and their relation to recovery criteria in 2842 patients followed over 3 years.** *Schizophr Res* 2009, **107:**165-172.

51. Lencucha R, Kinsella EA, Sumsion T: **The formation and maintenance of social relationships among individuals living with schizophrenia.** *Am J Psychiatr Rehabil* 2008, **11:**330-355.
52. Li Z, Arthur D: **Family education for people with schizophrenia in Beijing, China: randomised controlled trial.** *Br J Psychiatry* 2005, **187:**339-345.
53. Lincoln TM, Hodgins S: **Is lack of insight associated with physically aggressive behavior among people with schizophrenia living in the community?** *J Nerv Ment Dis* 2008, **196:**62-66.
54. Liu KWD, Hollis V, Warren S, Williamson DL: **Supported-employment program processes and outcomes: experiences of people with schizophrenia.** *Am J Occup Ther* 2007, **61:**543-554.
55. Magliano L, Fiorillo A, Malangone C, De Rosa C, Maj M, and the Family Intervention Working Group: **Patient functioning and family burden in a controlled, real-world trial of family psychoeducation for schizophrenia.** *Psychiatr Serv* 2006, **57:**1784-1791.
56. McCreadie RG, Kelly C, Connolly M, Williams S, Baxter G, Lean M, Paterson JR: **Dietary improvement in people with schizophrenia: randomised controlled trial.** *Br J Psychiatry* 2005, **187:**346-351.
57. Meijer CJ, Koeter MWJ, Sprangers MAG, Schene AH: **Predictors of general quality of life and the mediating role of health related quality of life in patients with schizophrenia.** *Soc Psychiatry Psychiatr Epidemiol* 2009, **44:**361-368.
58. Naber D, Riedel M, Klimke A, Vorbach EU, Lambert M, Kühn KU, Bender S, Bandelow B, Lemmer W, Moritz S, Dittmann RW: **Randomized double blind comparison of olanzapine vs. clozapine on subjective wellbeing and clinical outcome in patients with schizophrenia.** *Acta Psychiatr Scand* 2005, **111:**106–115.

59. Nathans-Barel I, Feldman P, Berger B, Modai I, Silver H: **Animal-assisted therapy ameliorates anhedonia in schizophrenia patients. A controlled pilot study**. *Psychother Psychosom* 2005, **74:**31-35.

60. Nolan KA, Shope CB, Citrome L, Volavka J: **Staff and patient views of the reasons for aggressive incidents: a prospective, incident-based study.** *Psychiatr Q* 2009, **80:**167-172.

61. Novick D, Haro JM, Suarez D, Vieta E, Naber D: **Recovery in the outpatient setting: 36-month results from the Schizophrenia Outpatients Health Outcomes (SOHO) study.** *Schizophr Res* 2009, **108:**223-230.

62. Olié JP, Spina E, Murray S, Yang R: **Ziprasidone and amisulpride effectively treat negative symptoms of schizophrenia: results of a 12-week, double-blind study.** *Int Clin Psychopharmacol* 2006, **21:**143–151.

63. Penadés R, Catalán R, Salamero M, Boget T, Puig O, Guarch J, Gastó C: **Cognitive Remediation Therapy for outpatients with chronic schizophrenia: a controlled and randomized study.** *Schizophr Res* 2006, **87:**323-331.

64. Puschner B, Angermeyer MC, Leese M, Thornicroft G, Schene A, Kikkert M, Burti L, Tansella M, Becker T: **Course of adherence to medication and quality of life in people with schizophrenia.** *Psychiatry Res* 2009, **165:**224-233.

65. Ramírez García JI, Chang CL, Young JS, López SR, Jenkins JH: **Family support predicts psychiatric medication usage among Mexican American individuals with schizophrenia.** *Soc Psychiatry Psychiatr Epidemiol* 2006, **41:**624-631.75.

66. Resnick SG, Rosenheck RA, Canive JM, De Souza C, Stroup TS, McEvoy J, Davis S, Keefe RSE, Swartz M, Lieberman J: **Employment outcomes in a randomized trial of second-generation antipsychotics and perphenazine in the treatment of individuals with schizophrenia.** *J Behav Health Serv Res* 2008, **35:**215-225.

67. Rice E: **The invisibility of violence against women diagnosed with schizophrenia: a synthesis of perspectives.** *Adv Nurs Sci* 2008, **31:**E9-E21.

68. Riedel M, Müller N, Strassnig M, Spellmann I, Engel RR, Musil R, Dehning S, Douhet A, Schwarz MJ, Möller HJ: **Quetiapine has equivalent efficacy and superior tolerability to risperidone in the treatment of schizophrenia with predominantly negative symptoms.** *Eur Arch Psychiatry Clin Neurosci* 2005, **255:**432–437.

69. Ritchie CW, Chiu E, Harrigan S, Macfarlane S, Mastwyk M, Halliday G, Hustig H, Hall K, Hassett A, O'Connor DW, Opie J, Nagalingam V, Snowdon J, Ames D: **A comparison of the efficacy and safety of olanzapine and risperidone in the treatment of elderly patients with schizophrenia: an open study of six months duration.** *Int J Geriatr Psychiatry* 2006, **21:**171-179.

70. Ritsner M, Gibel A, Ratner Y: **Determinants of changes in perceived quality of life in the course of schizophrenia.** *Qual Life Res* 2006, **15:**515-526.

71. Ritsner MS, Ratner Y: **The long-term changes in coping strategies in schizophrenia: temporal coping types.** *J Nerv Ment Dis* 2006, **194:**261-267.

72. Ritsner MS, Ratner Y, Gibel A, Weizman R: **Positive family history is associated with persistent elevated emotional distress in schizophrenia: evidence from a 16-month follow-up study.** *Psychiatry Res* 2007, **153:**217-223.

73. Russell TA, Chu E, Phillips ML: **A pilot study to investigate the effectiveness of emotion recognition remediation in schizophrenia using the micro-expression training tool.** *Br J Clin Psychol* 2006, **45:**579-583.

74. Ryu Y, Mizuno M, Sakuma K, Munakata S, Takebayashi T, Murakami M, Fallon IRH, Kashima H: **Deinstitutionalization of long-stay patients with schizophrenia:**

the 2-year social and clinical outcome of a comprehensive intervention program in Japan. *Aust N Z J Psychiatry* 2006, **40:**462-470.

75. Salokangas RKR, Honkonen T, Stengård E: **Social role behaviour of patients with long-term schizophrenia in the community during sharp decline in number of psychiatric beds.** *J Ment Health* 2007, **16:**663-678.
76. Salokangas RKR, Honkonen T, Stengård E, Koivisto AM: **Subjective life satisfaction and living situations of persons in Finland with long-term schizophrenia.** *Psychiatr Serv* 2006, **57:**373-381.
77. Sánchez P, Ojeda N, Peña J, Elizagárate E, Yoller AB, Gutiérrez M, Ezcurra J: **Predictors of longitudinal changes in schizophrenia: the role of processing speed.** *J Clin Psychiatry* 2009, **70:**888-896.
78. Schindler VP: **Role development: an evidenced-based intervention for individuals diagnosed with schizophrenia in a forensic facility.** *Psychiatr Rehabil J* 2005, **28:**391-394.
79. Schomerus G, Heider D, Angermeyer MC, Bebbington PE, Azorin JM, Brugha T, Toumi M: **Residential area and social contacts in schizophrenia. Results from the European Schizophrenia Cohort (EuroSC).** *Soc Psychiatry Psychiatr Epidemiol* 2007, **42:**617-622.
80. Schomerus G, Heider D, Angermeyer MC, Bebbington PE, Azorin JM, Brugha T, Toumi M: **Urban residence, victimhood and the appraisal of personal safety in people with schizophrenia: results from the European Schizophrenia Cohort (EuroSC).** *Psychol Med* 2008, **38:**591-597.
81. Seo JM, Ahn S, Byun EK, Kim CK: **Social skills training as nursing intervention to improve the social skills and self-esteem of inpatients with chronic schizophrenia.** *Arch Psychiat Nurs* 2007, **21:**317-326.

82. Siegel SJ, Irani F, Brensinger CM, Kohler CG, Bilker WB, Ragland JD, Kanes SJ, Gur RC, Gur RE: **Prognostic variables at intake and long-term level of function in schizophrenia.** *Am J Psychiatry* 2006, **163:**433-441.

83. Sim K, Chan YH, Chua TH, Mahendran R, Chong SA, McGorry P: **Physical comorbidity, insight, quality of life and global functioning in first episode schizophrenia: a 24-month, longitudinal outcome study.** *Schizophr Res* 2006, **88:**82-89.

84. Sirota P, Pannet I, Koren A, Tchernichovsky E: **Quetiapine versus olanzapine for the treatment of negative symptoms in patients with schizophrenia.** *Hum Psychopharmacol Clin Exp* 2006, **21:**227–234.

85. Soyka M, Graz C, Bottlender R, Dirschedl P, Schoech H: **Clinical correlates of later violence and criminal offences in schizophrenia.** *Schizophr Res* 2007, **94:**89-98.

86. Srinivasa Murthy R, Kishore Kumar KV, Chisholm D, Thomas T, Sekar K, Chandrashekar CR: **Community outreach for untreated schizophrenia in rural India: a follow-up study of symptoms, disability, family burden and costs.** *Psychol Med* 2005, **35:**341-351.

87. Strous RD, Ratner Y, Gibel A, Ponizovsky A, Ritsner M: **Longitudinal assessment of coping abilities at exacerbation and stabilization in schizophrenia.** *Compr Psychiatry* 2005, **46:**167-175.

88. Swartz MS, Perkins DO, Stroup TS, Davis SM, Capuano G, Rosenheck RA, Reimherr F, McGee MF, Keefe RSE: **Effects of antipsychotic medications on psychosocial functioning in patients with chronic schizophrenia: findings from the NIMH CATIE study.** *Am J Psychiatry* 2007, **164:**428–436.

89. Tabarés-Seisdedos R, Balanzá-Martínez V, Sánchez-Moreno J, Martinez-Aran A, Salazar-Fraile J, Selva-Vera G, Rubio C, Mata I, Gómez-Beneyto M, Vieta E:

Neurocognitive and clinical predictors of functional outcome in patients with schizophrenia and bipolar I disorder at one-year follow-up. *J Affect Disord* 2008, **109:**286-299.

90. Temple S, Ho BC: **Cognitive therapy for persistent psychosis in schizophrenia: a case-controlled clinical trial.** *Schizophr Res* 2005, **74:**195-199.
91. Thirthalli J, Venkatesh BK, Kishorekumar KV, Arunachala U, Venkatasubramanian G, Subbakrishna DK, Gangadhar BN: **Prospective comparison of course of disability in antipsychotic-treated and untreated schizophrenia patients.** *Acta Psychiatr Scand* 2009, **119:**209-217.
92. Usall J, Suarez D, Haro JM, the Soho Study Group: **Gender differences in response to antipsychotic treatment in outpatients with schizophrenia.** *Psychiatry Res* 2007, **153:**225-231.
93. Valencia M, Rascon ML, Juarez F, Murow E: **A psychosocial skills training approach in Mexican out-patients with schizophrenia.** *Psychol Med* 2007, **37:**1393-1402.
94. Vauth R, Corrigan PW, Clauss M, Dietl M, Dreher-Rudolph M, Stieglitz RD, Vater R: **Cognitive strategies versus self-management skills as adjunct to vocational rehabilitation.** *Schizophr Bull* 2005, **31:**55-66.
95. Villalta-Gil V, Roca M, Gonzalez N, Domènec E, Cuca, Escanilla A, Asensio MR, Esteban ME, Ochoa S, Haro JM, Schi-Can Group: **Dog-assisted therapy in the treatment of chronic schizophrenia inpatients.** *Anthrozoös* 2009, **22:**149-159.
96. Westheide J, Cvetanovska G, Albrecht C, Bliesener N, Cooper-Mahkorn D, Creutz C, Hornung WP, Klingmüller D, Lemke MR, Maier W, Schubert M, Sträter B, Kühn KU: **Prolactin, subjective well-being and sexual dysfunction: an open label**

observational study comparing quetiapine with risperidone. *J Sex Med* 2008, **5:**2816-2826.

97. Wölwer W, Frommann N, Halfmann S, Piaszek A, Streit M, Gaebel W: **Remediation of impairments in facial affect recognition in schizophrenia: efficacy and specificity of a new training program.** *Schizophr Res* 2005, **80:**295-303.
98. Wykes T, Hayward P, Thomas N, Green N, Surguladze S, Fannon D, Landau S: **What are the effects of group cognitive behaviour therapy for voices? A randomised control trial.** *Schizophr Res* 2005, **77:**201-210.
99. Xiang YT, Wang CY, Wang Y, Chiu HFK, Zhao JP, Chen Q, Chan SSM, Lee EHM, Ungvari GS: **Socio-demographic and clinical determinants of quality of life in Chinese patients with schizophrenia: a prospective study.** *Qual Life Res* 2010, **19:**317-322.
100. Xiang Y, Weng Y, Li W, Gao L, Chen G, Xie L, Chang Y, Tang WK, Ungvari GS: **Training patients with schizophrenia with the community re-entry module: a controlled study.** *Soc Psychiatry Psychiatr Epidemiol* 2006, **41:**464-469.
101. Xiang YT, Weng YZ, Li WY, Gao L, Chen GL, Xie L, Chang YL, Tang WK, Ungvari GS: **Efficacy of the Community Re-Entry Module for patients with schizophrenia in Beijing, China: outcome at 2-year follow-up.** *Br J Psychiatry* 2007, **190:**49-56.
102. Yilmaz M, Josephsson S, Danermark B, Ivarsson AB: **Participation by doing: social interaction in everyday activities among persons with schizophrenia.** *Scand J Occup Ther* 2008, **15:**162-172.
103. Yilmaz M, Josephsson S, Danermark B, Ivarsson AB: **Social processes of participation in everyday life among persons with schizophrenia.** *Int J Qual Stud Health Well-being* 2009, **4:**267-279.

104. Zhong KX, Sweitzer DE, Hamer RM, Lieberman JA: **Comparison of quetiapine and risperidone in the treatment of schizophrenia: a randomized, double-blind, flexible-dose, 8-week study.** *J Clin Psychiatry* 2006, **67:**1093-1103.

.

.
