# Supplementary material for: Disability and schizophrenia: a systematic review of experienced psychosocial difficulties
Source: BMC Psychiatry. 2012 Nov 9;12:193. doi: 10.1186/1471-244X-12-193 (PMC3539983; doi:10.1186/1471-244X-12-193)
Supplement: Additional file 3 — Table S1 Characteristics and main findings of included studies. [file 1471-244X-12-193-S3.pdf]

Additional File 3

Supplementary Table S1 Characteristics and main findings of included studies

Reference	Country	Study design	Follow-up	Intervention	Participants	Aims	Main findings
1. (Addington et al., 2006)	Canada	longitudinal observational study	1 year	n/a	53 patients with chronic schizophrenia, 50 patients with first-episode psychosis, 55 non-psychiatric controls	To test the hypothesis that facial affect recognition is a mediator between cognitive and social functioning.	Both the first-episode and multi-episode groups were found to be impaired relative to non-psychiatric controls in cognition, social functioning and facial affect recognition. There were significant associations among facial affect recognition, cognition and social functioning in all three groups. For the patient groups, but not healthy controls, there was evidence that facial affect recognition did partially mediate the relationship between cognitive and social functioning.
2. (Addington et al., 2006)	Canada	longitudinal observational study	1 year	n/a	53 patients with chronic schizophrenia, 50 patients with first-episode psychosis, 55 non-psychiatric controls	To test the hypotheses that (a) deficits in social perception were associated with deficits in social functioning and cognition and (b) that social perception mediated between cognitive and social functioning.	People with schizophrenia were found to have deficits in social knowledge and social perception relative to healthy controls. These deficits remained stable over time in both patient groups. There were significant associations between social cognition, cognition and social functioning in all three groups. The study findings also provide some evidence that social cognition mediates the relationship between cognitive and social functioning.
3. (Alonso et al., 2009)	10 European countries	longitudinal observational study	3 years	n/a	9340 patients with schizophrenia	To describe the evolution of health-related quality of life (HRQL) in patients with schizophrenia and to investigate the association between continuous antipsychotic use and HRQL.	Antipsychotic treatment over a 36-month period was associated with substantial and sustained improvement in HRQL. The largest improvement occurred in the first six months. There were few differences in HRQL change between medication groups. Shorter duration of illness and earlier age at

Reference	Country	Study design	Follow-up	Intervention	Participants	Aims	Main findings
							first treatment were associated with better HRQL outcomes, as were being in paid employment or being more socially active at study entry.
4. (Alptekin et al., 2005)	Turkey	longitudinal observational study	1 year	n/a	382 patients with schizophrenia	To assess the level of disability in patients with schizophrenia and to identify its clinical correlates and predictors.	Total disability scores were significantly decreased at follow-up. Disability was positively correlated with psychopathology and with medication side-effects. Patients with the disorganized subtype of schizophrenia and residual symptoms were more disabled than patients with other subtype diagnoses. Negative symptoms and duration of untreated psychosis were significant predictors of disability after 1 year. Early-onset schizophrenia had a twofold increased risk for developing disability.
5. (Álvarez et al., 2006)	Spain	randomized controlled trial	1 year	olanzapine vs. risperidone	250 patients with schizophrenia	To evaluate the efficacy of olanzapine compared with risperidone in negative symptoms in schizophrenic outpatients with prominent negative symptoms.	Both agents significantly improved psychopathology. However, olanzapine patients showed higher improvement than risperidone patients on the Scale for the Assessment of Negative Symptoms (SANS) summary score and on the affective flattening and avolition/apathy SANS subscales. There were also significant improvements in favor of olanzapine in positive symptoms and social functioning. Olanzapine patients reported less extrapyramidal side effects but a higher incidence of body weight increase.
6. (Arango et al., 2006)	Spain	randomized controlled trial	1 year	oral vs. depot formulation of zuclopenthixol	46 patients with schizophrenia	To longitudinally assess the effect of depot and oral medication on treatment compliance, and the effect of treatment compliance on	Physical aggression among previously violent schizophrenia patients during the follow-up year was inversely proportional to treatment adherence, better compliance, and greater

Reference	Country	Study design	Follow-up	Intervention	Participants	Aims	Main findings
						reduction of violence.	reduction of positive symptoms. Lower frequency of violent acts was observed in subjects receiving depot medication. Regardless of route of administration, treatment non-adherence was the best predictor of violence.
7. (Beebe et al., 2008)	USA	randomized controlled trial	3 months	telephone intervention problem solving (TIPS) vs. treatment as usual (TAU)	29 patients with schizophrenia	To evaluate the effectiveness of telephone intervention problem solving (TIPS) in improving medication adherence.	Participants receiving TIPS had significantly higher adherence to psychiatric medications throughout the study period. No difference was found between the experimental and control groups with regard to adherence to non-psychiatric medications.
8. (Beebe et al., 2005)	USA	randomized controlled trial	16 weeks	treadmill exercise program vs. control (non-intervention) group	10 patients with schizophrenia	To compare the physical and mental health parameters of patients with schizophrenia participating in a structured 16-week treadmill walking program with those of a control group from the same population not participating in the exercise program during the same time period.	Participants in the walking group experienced significant reductions in body fat compared to a control group. Experimental participants also had greater aerobic fitness, lower BMI, and fewer psychiatric symptoms than controls at the conclusion of the program, but these differences did not reach statistical significance.
9. (Bejerholm & Eklund, 2006)	Sweden	qualitative study	n/a	n/a	20 patients with schizophrenia	To provide insight into how people with schizophrenia engage in daily occupations, interact with their environment, and endeavour to make sense of their experiences.	Participants showed considerable variation in the levels of engagement in daily occupations. Each level of occupational engagement was related to a daily rhythm and a sense of meaning. Levels of engagement ranged from performing mostly quiet activities, alone, with little sense of meaning, to engaging in meaningful occupations that involved social interactions. In general, female participants preferred activities in their home environment, while males preferred activities outside their home environment.

Reference	Country	Study design	Follow-up	Intervention	Participants	Aims	Main findings
10. (Bitter et al., 2005)	27 countries	longitudinal observational study	6 months	n/a	570 patients with schizophrenia	(1) To examine sexual functioning among first-time treated schizophrenia patients at the time that they initiated antipsychotic treatment, and again 3 and 6 months later. (2) To compare the impact of olanzapine, risperidone and typical antipsychotics on sexual functioning.	Before starting antipsychotic treatment, 20.2% of the patients reported being unable to perform sexually, 16.8% reported some problems in sexual functioning and 62.9% reported no problems. After being treated, patients receiving olanzapine showed the lowest prevalence of neuroleptic-related loss of libido and sexual dysfunction.
11. (Borras et al., 2007)	Switzerland	longitudinal observational study	1 month	n/a	57 patients with schizophrenia	To describe how patients with schizophrenia spend their money.	Participants devoted 39% of their discretionary money on psychoactive substances (cigarettes, alcohol, cannabis), 33% on various drinks in coffee houses, and 28% on leisure activities. The way patients spent this money appeared to be relatively independent of their clinical and socio-demographic characteristics (including neuropsychological measures). The only relevant, significant characteristic linked to greater spending on alcohol and cannabis was an earlier age at the first hospitalization.
12. (Breier et al., 2005)	12 sites in Europe and North and South America	randomized controlled trial	28 weeks	olanzapine vs. ziprasidone	548 patients with schizophrenia	To compare the efficacy and safety of olanzapine and ziprasidone.	Olanzapine was significantly superior to ziprasidone on the primary efficacy measure of the PANSS total score. In addition, significantly greater improvements for the olanzapine group were found on the PANSS subscales for positive symptoms, negative symptoms, general psychopathology, cognition, and excitability and on the CGI improvement and severity scales. Furthermore, in the analysis using the last observation carried forward, greater improvements in depressive and

Reference	Country	Study design	Follow-up	Intervention	Participants	Aims	Main findings
							anxiety symptoms in the olanzapine group were found, as determined with the Montgomery-Åsberg Depression Rating Scale and Hamilton anxiety scale, respectively. Ziprasidone was superior for weight change and lipid profile.
13. (Buizza et al., 2007)	Italy	qualitative study	n/a	n/a	26 patients with schizophrenia, 22 relatives	To explore the stigma and identify its constituent elements from the perspective of people with schizophrenia and their relatives in a local Italian context, in order to collect concrete suggestions for stigma-reduction interventions.	The sample reported 428 concrete cases of stigmatization: 198 experiences were described by patients and 230 by relatives. From the patient and relative group statements four dimensions of stigma were identified: (1) access to social roles, (2) internalization of stigma, (3) quality of mental health services, and (4) public image of mental illness.
14. (Cavallaro et al., 2009)	Italy	randomized controlled trial	12 weeks	standard rehabilitation treatment (SRT) + computer-aided cognitive remediation treatment (CRT) vs. SRT + placebo training	86 patients with schizophrenia	To assess the effectiveness of intensive computer-aided cognitive remediation treatment (CRT) added to a standard rehabilitation treatment (SRT) in enhancing neuropsychological performances and daily functioning in patients with schizophrenia.	The repeated measures ANOVA showed a significant differential improvement in cognitive flexibility and attention performances and quality of life measures in favour of the SRT + CRT group. Also the effect sizes of improvement, calculated post hoc in order to compare the magnitude of improvement between groups, were significantly higher in the intervention group.
15. (Ceccato et al., 2006)	Italy	controlled clinical trial	18 weeks	Sound Training for Attention and Memory (STAM) vs. improvisational music therapy	16 patients with schizophrenia	To appraise the possible effects of a specific music therapy protocol (STAM) on specific components of attention and memory.	The patients who participated in the experimental music therapy activity showed a significantly improved performance on measures of memory and social disability. The control group showed improvement only in the domain of social disability. No significant changes in attention were noted in both groups.

Reference	Country	Study design	Follow-up	Intervention	Participants	Aims	Main findings
16. (Chang et al., 2009)	South Korea	longitudinal observational study	6 months	n/a	40 patients with schizophrenia (21 first-episode schizophrenia patients and 19 chronic schizophrenia patients)	To investigate the longitudinal changes in the phenomenological dimensions of auditory verbal hallucinations (AVHs) in patients diagnosed with schizophrenia.	The phenomenological variables of AVHs formed two main clusters with 6 months treatment: emotional and cognitive. Physical characteristics of AVHs (i.e. frequency, loudness, and duration) failed to indicate a stable cluster. Two structural changes occurred from baseline to 6 month follow-up: "frequency" and "disruption to life caused by voices" moved from the cognitive to the emotional domain. The first-episode group showed more dynamic changes than did that of the chronic schizophrenia group.
17. (Chien et al., 2006)	Hong Kong	randomized controlled trial	6-month intervention, 18-month follow-up	mutual support group for families of people with schizophrenia vs. psychoeducation vs. standard care	97 family carers, 96 patients with schizophrenia	To examine the effects of a mutual support group for families of people with schizophrenia, compared with psychoeducation and standard care.	Mutual support consistently produced greater improvement in patient and family functioning and caregiver burden over the intervention and follow-up periods, compared with the other two conditions. The psychoeducation group also demonstrated a significant and consistent improvement in patient functioning compared with standard care. Whereas the total number of patients’ readmissions to hospital did not differ between the three groups, the participants in the mutual support group reported a greater reduction in the duration of readmissions than the other two groups.
18. (Chien et al., 2008)	Hong Kong	randomized controlled trial	6-month intervention, 12-month post-intervention follow-up	peer-led mutual support group for families of people with schizophrenia vs. standard psychiatric care	76 family carers, 76 patients with schizophrenia	To evaluate the effectiveness of a family-led mutual support group for caregivers of schizophrenia sufferers compared with standard psychiatric care.	The mutual support group experienced significantly greater improvements in families' burden, functioning and number of support persons and length of patients' re-hospitalizations at two post-tests (at one week and 12 months following completion of the

Reference	Country	Study design	Follow-up	Intervention	Participants	Aims	Main findings
							intervention). In both groups, no significant changes were found in demands for family support services and severity of patients' psychiatric symptoms over the 12-month follow-up period.
19. (Chien et al., 2007)	Hong Kong	randomized controlled trial	36-week intervention, 12-month post-intervention follow-up	family psychoeducation group program vs. standard care	84 family members, 84 patients with schizophrenia	To test the effectiveness of a family psychoeducation group program for people with schizophrenia compared with standard care.	Participants in the psychoeducation group reported greater improvements in families' and patients' functioning, families' burden of care, and the number and length of patients' re-hospitalizations over the 12-month follow-up period, compared with the standard care group.
20. Chrzanowski et al., 2006)	USA, Czech Republic, Poland, Russia, Ukraine	randomized controlled trial	52 weeks	aripiprazole vs. olanzapine	214 patients with schizophrenia	To compare the long-term efficacy and safety of aripiprazole with olanzapine in patients with either acute relapsing or chronic, stable schizophrenia.	For patients with chronic stable illness, both aripiprazole and olanzapine maintained symptom control, providing similar, modest improvements in efficacy scores. Patients who completed the treatment period experienced a mean improvement in PANSS Total score of approximately 10% from baseline. Among the patients experiencing acute relapse of schizophrenia, aripiprazole, and olanzapine treatment provided similar, sustained improvements in symptom scores over the study period. The improvements in efficacy scores were larger in these more severely ill patients than in chronic stable patients, with a mean improvement in PANSS Total score from baseline of approximately 30% among completers. Aripiprazole demonstrated a superior safety/tolerability profile to olanzapine.

Reference	Country	Study design	Follow-up	Intervention	Participants	Aims	Main findings
21. (Conley et al., 2005)	USA	randomized controlled trial	12 weeks	risperidone vs. quetiapine vs. fluphenazine	38 patients with schizophrenia	To evaluate the effectiveness of risperidone, quetiapine, or fluphenazine in a stringently defined treatment-resistant population of people with schizophrenia.	No differences were noted in symptom changes during 12 weeks of treatment with risperidone, quetiapine, or fluphenazine. The majority of patients improved only minimally to moderately, and most patients continued to have substantial residual symptoms. There were also no significant differences in side effects.
22. (Dossenbach et al., 2006)	27 countries	longitudinal observational study	1 year	n/a	3828 patients with schizophrenia	To compare the frequency of sexual dysfunction in patients with schizophrenia who were treated with selected atypical or typical antipsychotic agents in a naturalistic, clinical practice setting.	Almost half of the monotherapy patients reported loss of libido at enrolment, and approximately 30% of patients reported impotence or sexual dysfunction. Monotherapy with the atypical agents olanzapine and quetiapine was associated with fewer problems with sexual function than treatment with the prolactin-elevating risperidone, or the typical agent haloperidol. There were significant discrepancies between patient and psychiatrist perception of the presence of adverse events related to sexual dysfunction, with psychiatrists underestimating sexual dysfunction.
23. (Fisher et al., 2009)	USA	randomized controlled trial	10 weeks	computerized auditory training program vs. control condition of commercial computer games	55 patients with schizophrenia	To investigate the effectiveness of an innovative, neuroplasticity-based, cognitive training program that targets both early auditory processing and working memory operations, with the ultimate goal of improving verbal memory performance in schizophrenia.	Relative to the control group, patients who received active training showed significant gains in global cognition, verbal working memory, and verbal learning and memory. They also showed significant improvement in auditory psychophysical performance; this improvement was significantly correlated with gains in verbal working memory and global cognition. As regards the effect sizes, strong positive effects on verbal cognition measures were found for the training condition,

Reference	Country	Study design	Follow-up	Intervention	Participants	Aims	Main findings
							but with no difference between conditions in effect on visual cognition, indicating the targeted nature of the training approach.
24. (Fuentes et al., 2007)	Spain	randomized controlled trial	3-month intervention, 6-month post-intervention follow-up	social perception training vs. standard care	18 patients with schizophrenia	To investigate the effectiveness of social perception training in patients with schizophrenia, and to test the possible link between cognition, social cognition, and functional outcome in schizophrenia.	The patients that were trained improved their social perception abilities relative to the control group. There were no differences between groups with respect to psychopathology, attention, and social functioning. The data did not show the expected link between non-social cognition, and functional outcome through social cognition as a mediator variable.
25. (Gelkopf et al., 2006)	Israel	controlled clinical trial	3 months	watching humorous movies vs. watching neutral movies	29 patients with schizophrenia	To assess the impact of humorous movies on psychopathology, anxiety, depression, anger, social functioning, insight, and therapeutic alliance.	The use of humorous movies reduced psychopathology, and more specifically negative symptomatology, as well as anxiety and depression. There was also less self-reported anger and an improvement in social functioning. No changes were observed in treatment insight or working alliance.
26. (Gil Sanz et al., 2009)	Spain	randomized controlled trial	10 weeks	Social Cognition Training Program (SCTP) vs. standard care	14 patients with schizophrenia	To evaluate the efficacy of the Social Cognition Training Program (SCTP), a program that includes emotion recognition training and social perception training.	The experimental group members improved their perception and interpretation of social situations, but not their emotion recognition. An improvement was also observed in the areas of personal care and daily activities. The intervention did not produce any significant change in attention or psychopathology.
27. (González-Torres et al., 2007)	Spain	qualitative study	n/a	n/a	18 patients with schizophrenia, 26 relatives	To explore actual stigma and discrimination experience recalled by people with schizophrenia and their relatives, and reflect on the consequences of those experiences on	The following categories of stigma and discrimination experiences were extracted from the patients' data:<br>(1) Mental illness vs. Lack of will,<br>(2) Prejudice related to dangerousness,<br>(3) Over-protection – Infantilization,<br>(4) Daily social discrimination,

Reference	Country	Study design	Follow-up	Intervention	Participants	Aims	Main findings
						patients, relatives, and society in general.	(5) Discrimination in health care,<br>(6) Descendants,<br>(7) Avoidance – Social isolation.<br>Data from relatives were divided into three sets: (1) Discrimination towards the patients witnessed by relatives, (2) Discrimination suffered by the relatives themselves and (3) Discrimination exerted by the relatives on the patients.
28. (Górna et al., 2008)	Poland	longitudinal observational study	4-6 years	n/a	74 patients with schizophrenia	(1) To evaluate changes in objective and subjective quality of life (QoL) of first-time-admission patients with schizophrenia. (2) To identify determinants of QoL at different time points and assess stability of the set of determinants over the course of several years. (3) To check for changes in the psychiatric symptoms over time.	The objective and subjective QoL of the participants was relatively low. Over time, the objective QoL remained stable in three domains (social engagement/withdrawal, prosocial activities, and independence-competence), improved in three domains (independence-performance, recreation/pastimes, and interpersonal behavior), and decreased in one domain (employment/occupation). The subjective QoL did not change. A significant deterioration was observed for all groups of symptoms but one (cognitive). The most important predictors of both dimensions of QoL were psychopathology and duration of untreated psychosis.
29. (Gray et al., 2006)	Netherlands, Germany, England, Italy	randomized controlled trial	1 year	adherence therapy vs. health education control intervention	409 patients with schizophrenia	To evaluate the effectiveness of adherence therapy in improving treatment adherence and clinical outcomes for people with schizophrenia.	There were no significant differences in quality of life between adherence therapy and health education groups at baseline or at follow-up. There was also no significant difference in medication adherence between the two groups at follow-up. Finally, the experimental and control groups did not differ significantly at baseline or at follow-up in terms of psychopathology.

Reference	Country	Study design	Follow-up	Intervention	Participants	Aims	Main findings
30. (Grover et al., 2005)	India	economic evaluation	6 months	n/a	50 patients with schizophrenia, 50 patients with diabetes mellitus	To examine the costs of care of schizophrenia in a developing setting (India), to compare this with an equivalent group of patients with diabetes mellitus, and to assess the influence of certain demographic, social and clinical variables on costs of treatment.	Total costs of care over 6 months were more for diabetes mellitus (145 USD) than schizophrenia (137 USD), but this difference was not significant. Indirect costs (63%) made up the major proportion of total treatment costs for schizophrenia, while direct costs accounted for only a third of the total. The opposite was true for diabetes. Healthcare provider costs constituted only negligible proportions of total costs of care, for both schizophrenia and diabetes mellitus. Expenses on drugs, travel, and visits to doctors or native healers, etc. made up the bulk of the patient and family costs. These costs were significantly more for diabetes. Total treatment costs in schizophrenia were significantly higher in those who were unemployed, those who visited the hospital more often, and were more severely ill and disabled.
31. (Gur et al., 2006)	USA	longitudinal observational study	1 year	n/a	162 patients with schizophrenia (63 patients with flat affect and 99 patients without flat affect), 138 healthy controls	To compare patients with schizophrenia with flat affect with those without flat affect in three domains: clinical, emotion processing, and neurocognitive.	Patients with schizophrenia, compared with controls, were impaired on facial emotion processing tasks, one that required identification of happy and sad emotions and another that required differentiating among intensities within these emotions. They responded inaccurately yet faster than controls for the intensity differentiation task, suggesting a decomposition of the normal relation between accuracy and speed. Flat affect was more common in men and was associated with poorer premorbid adjustment, worse current quality of life, and worse outcome at

Reference	Country	Study design	Follow-up	Intervention	Participants	Aims	Main findings
							1-year follow-up. Flat affect ratings, compared with other negative symptoms, uniquely predicted performance on emotion processing tasks. Patients with flat affect showed greater impairment in both emotion processing tasks, with the most pronounced impairment for the intensity differentiation task. However, the two patient groups (Flat Affect group and Non-Flat Affect group) did not differ in the neurocognitive profile except for verbal memory.
32. (Harrow et al., 2005)	USA	longitudinal observational study	15 years	n/a	64 patients with schizophrenia, 12 patients with schizophreniform disorders, 81 patients with other psychotic disorders, 117 nonpsychotic control patients	To study course, outcome, and potential recovery in a sample of patients with schizophrenia, and in control samples of psychotic and nonpsychotic patients.	By the 15-year follow-up, 41% of the patients with schizophrenia showed 1 or more periods of recovery at some point. However, at each of the 5 follow-ups over the 15-year period they showed poorer mean global outcome scores than the other 3 groups. Most schizophrenia patients did not show the severe social isolation. Over 50% of the schizophrenia patients did not have a disorder that was chronic and continuous. Rather, their disorder was episodic.
33. (Heider et al., 2007)	UK, France, Germany	longitudinal observational study	2 years	n/a	1208 patients with schizophrenia	To identify factors influencing the subjective quality of life (QoL) of patients with schizophrenia	Subjective QoL of schizophrenia patients proved to be related to objective QoL, illness symptoms, socio-demographic characteristics and country of residence. The scores in several domains of subjective QoL (living situation, daily activities and functioning, family, legal and safety issues, health) were significantly lower in the UK than in Germany. Differences between France and Germany were less pronounced, with

Reference	Country	Study design	Follow-up	Intervention	Participants	Aims	Main findings
							significantly less satisfaction with living situation and family in France. Objective QoL scores were generally found to be related to the corresponding subjective QoL scores, but sometimes also to the respondents' perception of other domains. Patients' financial situation, and depressive and positive symptoms had a general effect on almost all subjective domains. QoL was more strongly related to time-invariant differences between individuals than to intra-personal changes over time.
34. (Herbener et al., 2005)	USA	longitudinal observational study	on average 31.3 days	n/a	13 patients with schizophrenia, 13 healthy comparison subjects	(1) To evaluate emotion perception in acutely ill patients experiencing a first episode of schizophrenia. (2) To investigate the effects of antipsychotic medication on emotion perception.	At the baseline assessment, patients with schizophrenia performed more poorly than healthy controls on both the emotion acuity task and the emotion differentiation task. There was a robust decrease in symptom scores in the patient group from baseline to follow-up. However, clinical improvement was unrelated to change in emotion task performance. There was no significant improvement on any of the emotional processing tasks after treatment. Emotion perception deficits were correlated with negative symptoms after clinical stabilization.
35. (Hill et al., 2010)	Australia	qualitative study	n/a	n/a	7 patients with schizophrenia	To investigate the process of transition to independent accommodation for people with schizophrenia.	The analysis of the data revealed that the transition to independent accommodation involved three processes which did not occur in a linear or sequential progression but rather developed simultaneously: (1) developing a sense of control, (2) establishing a relationship between illness and place, and (3) attaining a sense of belonging.

Reference	Country	Study design	Follow-up	Intervention	Participants	Aims	Main findings
36. (Honkonen et al., 2007)	Finland	longitudinal retrospective study	3 years	n/a	2168 patients with schizophrenia (three cohorts of patients, who were discharged from psychiatric hospitals in 1986, 1990, and 1994)	To investigate the competitive employment rate and predictors of competitive employment in a large and unselected nationwide sample of discharged schizophrenia patients during an era of rapid deinstitutionalization in Finland.	Altogether, an average of 12.0% of the study population was either competitively or non-competitively employed 3 years after discharge. At follow-up, the competitive employment rate declined among the three cohorts, whereas the rate of non-competitive employment remained at the same level. The probability of competitive employment was higher for those who, at the time of discharge, were not on disability pension and had a current or past history of marriage, and had been discharged in the late 1980s. The probability of being competitively employed was lower for those who had no occupation at discharge and for those who had spent more time in hospital care during the three years after discharge.
37. Horan et al., 2007)	USA	longitudinal observational study	5 weeks	n/a	85 patients with schizophrenia, 18 patients with bipolar disorder, 18 healthy controls	To evaluate the stress and coping responses of schizophrenia patients and two comparison samples of bipolar patients and healthy controls following exposure to a natural disaster	Both schizophrenia and bipolar patients reported higher avoidance symptoms than controls one week after the earthquake. At the 5-week follow-up, the schizophrenia group had lower approach coping, self-esteem, and social support than controls, with scores in the bipolar group tending to fall between these groups. Within the schizophrenia group, higher levels of avoidance coping predicted higher residual stress symptoms at follow-up.
38. (Inadomi et al., 2005)	Japan	longitudinal observational study	3 years	n/a	28 patients with schizophrenia	To examine the efficacy of a 3-year psychiatric daycare (DC) program with regard to psychiatric symptoms and difficulties with daily living.	After 3 years of DC program, no significant changes were found in the severity of psychopathological symptoms. However, over this period, significant improvements were observed in the domains of daily living,

Reference	Country	Study design	Follow-up	Intervention	Participants	Aims	Main findings
							interpersonal relations, expressiveness, communication, initiative within a group, and cooperation in work tasks.
39. Jockers-Scherübl et al., 2005)	Germany	randomized controlled trial	12 weeks	paroxetine vs. placebo as an adjunct to antipsychotic treatment	29 patients with schizophrenia	To evaluate the effect of paroxetine as augmentation to antipsychotics in the treatment of negative symptoms in chronic schizophrenia.	There was a greater reduction in the PANSS negative subscale scores during treatment with paroxetine compared to placebo. When single items of the negative subscale of the PANSS were analyzed, there was a significant improvement in affective blunting, impaired abstract thinking and lack of spontaneity and flow of conversation in the paroxetine group compared to the placebo group. There was no significant difference between the groups with respect to emotional withdrawal, passive social withdrawal and stereotyped thinking. The Hamilton depression score remained practically unchanged in the low range in both groups. No significant side-effects occurred.
40. (Kallert et al., 2007)	Germany	longitudinal observational study	2 years	n/a	244 patients with schizophrenia; five subgroups living in (1) a psychiatric nursing home (n=50), (2) in social therapeutic hostels (n=51), (3) in sheltered community residential care (n=38), (4) at home with family (n=50) and (5) alone in their own homes (n=55)	To assess the effects of different types of community-based supported housing facilities for people with schizophrenia on psychopathological symptoms, social disabilities, subjective quality of life, and the normative individual needs for care.	In general, changes in the outcome parameters within the assessed 2-year period were rather small for all subgroups from a clinical perspective, demonstrating deterioration, particularly for the older and more severely disabled group living in the nursing home. The only statistically significant improvement of psychopathological symptoms within the five groups assessed appeared in the area of positive symptoms among patients living in social therapeutic hostels. Results from linear variance analytical statistical models revealed that group assignment played a

Reference	Country	Study design	Follow-up	Intervention	Participants	Aims	Main findings
							significant role only for the baseline-controlled development of social disabilities and subjective quality of life. Contrasts between the groups showed the disadvantage of nursing homes and hostels in terms of subjective quality of life and the advantage of staying integrated into family life in terms of social disabilities.
41. (Kane et al., 2006)	India	phase 1: prospective open-label treatment with haloperidol; phase 2: nonresponders were randomized to double-blind treatment with ziprasidone or chlorpromazine	6 weeks (phase 1); 12 weeks (phase 2)	ziprasidone vs. chlorpromazine	415 patients with schizophrenia in phase 1; 306 treatment-resistant patients with schizophrenia in phase 2	To evaluate the efficacy and safety of ziprasidone and chlorpromazine in patients who had a history of treatment-refractory schizophrenia and who were resistant to treatment with haloperidol.	Ziprasidone was more effective than chlorpromazine in improving negative symptoms and comparable to chlorpromazine in improving overall psychotic symptoms and global illness severity. Both ziprasidone- and chlorpromazine-treated subjects showed improvement in depressive symptoms. The most common treatment-related, treatment-emergent adverse events in both therapeutic groups were akathisia, dizziness, extrapyramidal symptoms, somnolence and tardive dyskinesia. Ziprasidone was associated with a greater decrease in median prolactin levels and a lower incidence of clinically significant weight change.
42. (Kane et al., 2007)	USA, Canada	phase 1: open-label treatment with olanzapine or risperidone; phase 2: nonresponders were randomized to double-blind treatment with aripiprazole or	4-6 weeks (open-label phase); 6 weeks (double-blind phase)	aripiprazole vs. perphenazine	416 patients with schizophrenia in phase 1; 300 treatment-resistant patients with schizophrenia in phase 2	To compare the efficacy and safety of aripiprazole and perphenazine in treatment-resistant patients with schizophrenia.	Treatment with either aripiprazole or perphenazine was associated with clinically relevant improvements in PANSS total scores. Overall, 27% of aripiprazole-treated patients and 25% of perphenazine-treated patients were classified as treatment responders, according to CGI or PANSS measures after 6 weeks. The mean changes in Quality of Life Scale (QLS) score from

Reference	Country	Study design	Follow-up	Intervention	Participants	Aims	Main findings
		perphenazine					baseline were similar in both groups, with no between-group statistical significance. The proportion of patients experiencing a clinically important improvement in quality of life was higher in the aripiprazole group (36%) than in the perphenazine group (21%); this difference approached statistical significance. The most frequently reported adverse events were insomnia, agitation, and anxiety in the perphenazine group and insomnia, agitation, and headache in the aripiprazole group. Perphenazine-treated patients had a higher incidence of extrapyramidal symptom–related adverse events.
43. (Karow et al., 2007)	Germany	longitudinal observational study	1 year	n/a	2960 patients with schizophrenia	To investigate longitudinally the associations of subjective well-being, clinical symptoms and side effects with compliance with antipsychotic treatment.	At the 12-month visit, 82.9% of the participants were classified as compliant by the physician, and 88.3% rated themselves as compliant. Compliance with antipsychotic medication was strongly associated with changes in patients' subjective well-being. Results showed a marked improvement in subjective well-being for patients with an increase in compliance and only a minor improvement in patients with a decrease in compliance during the observation period. Considering the impact of all Subjective Well-being under Neuroleptic Treatment (SWN-K) subscales, self-control showed the highest association with compliance. Apart from subjective well-being, positive symptoms at baseline and changes in positive symptoms were

Reference	Country	Study design	Follow-up	Intervention	Participants	Aims	Main findings
							found to have the strongest impact on compliance. Overall, side effects showed a moderate correlation with compliance, whereas changes in extrapyramidal symptoms demonstrated the strongest association.
44. (Keefe et al., 2007)	USA	randomized controlled trial	18 months	olanzapine vs. quetiapine vs. risperidone vs. ziprasidone vs. perphenazine	817 patients with schizophrenia	To compare the neurocognitive effects of olanzapine, perphenazine, quetiapine, risperidone, and ziprasidone in patients with chronic schizophrenia.	At 2 months, treatment resulted in small neurocognitive improvements for all antipsychotic agents, with no significant differences between groups. Results at 6 months were similar. After 18 months of treatment, neurocognitive improvement was greater in the perphenazine group than in the olanzapine and risperidone groups. Neurocognitive improvement predicted longer time to treatment discontinuation, independently from symptom improvement, in patients treated with quetiapine or ziprasidone.
45. (Kolotkin et al., 2008)	12 European countries	randomized controlled trial	26 weeks	aripiprazole vs. standard of care	555 patients with schizophrenia	To examine changes in weight and weight-related quality of life among community patients with schizophrenia treated with aripiprazole versus standard of care, consisting of other atypical antipsychotics (olanzapine, quetiapine, and risperidone).	Compared to standard of care, patients treated with aripiprazole experienced decreased weight and reported significantly greater improvements in physical function, self-esteem, sexual life, and overall weight-related quality of life. A clinically meaningful change in weight was associated with a meaningful change in quality of life.
46. (Konarzewska et al., 2009)	Poland	controlled clinical trial	8 weeks	risperidone vs olanzapine	89 male patients with schizophrenia	(1) To explore the effect of risperidone and olanzapine on the hypothalamo-pituitary-gonadal (HPG) axis in male patients with schizophrenia. (2) To identify the associations between the endocrine	Risperidone and olanzapine showed marked differences in their effects on pituitary and gonadal hormone levels. There were no statistically significant between-group differences in the intensity of psychopathological symptoms. The non-adherence to treatment and sexual dysfunction were

Reference	Country	Study design	Follow-up	Intervention	Participants	Aims	Main findings
						actions of the two medications and sexual dysfunction, treatment adherence and psychopathology.	significantly more severe in patients receiving risperidone. Sexual dysfunction and medication non-adherence was not related to prolactin or gonadal hormone levels.
47. (Kulhara et al., 2009)	India	randomized controlled trial	9 months	structured psychoeducational intervention for caregivers vs. routine outpatient care	76 patients with schizophrenia, 76 caregivers	To evaluate the impact of a structured psychoeducational intervention for caregivers of patients with schizophrenia on several patient- and caregiver-related parameters including severity of illness, levels of disability, caregiver burden and coping, and perceived social support and satisfaction with treatment among caregivers.	Structured psychoeducational intervention was significantly better than routine out-patient care in the areas of amelioration of psychopathology (all subscales of the PANSS), reduction of disability levels, increased perception of social support and increased satisfaction with treatment among caregivers. However, it did not significantly reduce drop-out, relapse and caregiver-burden, or improve caregiver-coping.
48. (Lambert et al., 2008)	Germany	longitudinal observational study	3 years	n/a	392 patients with schizophrenia	To identify remission and recovery rates as well as their predictors in schizophrenia.	At 3-year follow-up, remission rates for symptoms, functioning and subjective wellbeing were 60.3%, 45.4% and 57.0%; recovery rates were 51.7%, 35.0% and 44.3%. Of those, 28.1% were in combined remission and 17.1% in combined recovery. Remission and recovery were mainly predicted by the functional status at baseline and early remission of all three outcome measures at 3 months.
49. (Lambert et al., 2006)	Germany	longitudinal observational study	2 years	n/a	2960 patients with schizophrenia	To assess rates and predictors of symptomatic and functional remission as well as adequate subjective well-being/quality of life in a large cohort of patients with schizophrenia.	At endpoint, 47.2% of the patients achieved symptomatic remission, 26.6% achieved functional remission, and 42.2% achieved adequate subjective wellbeing. At endpoint, 12.8% were in complete remission. In 35.1% of the patients, none of the 3 remission criteria were achieved. Each

Reference	Country	Study design	Follow-up	Intervention	Participants	Aims	Main findings
							single remission component as well as complete remission was mainly predicted by early remission within the first 3 months. First-line treatment with atypical antipsychotics increased the likelihood of complete remission compared to conventional antipsychotics.
50. (Lambert et al., 2009)	Germany	longitudinal observational study	3 years	n/a	2842 patients with schizophrenia	(1) To study the longitudinal patterns of subjective wellbeing in schizophrenia using cluster analysis and their relation to recovery criteria. (2) To examine predictors for cluster affiliation. (3) To evaluate the sensitivity and specificity of baseline subjective wellbeing cut-offs for cluster affiliation.	Four clusters of patients with different subjective wellbeing patterns were identified: a stable low (33%), a stable moderate (31%), a stable high (16%), and a cluster with distinct initial improvement and then stable high subjective wellbeing (20%). These clusters were highly concordant with 3-year patterns of symptoms, quality of life, and, in part, psychosocial functioning. Stable low cluster affiliation was mainly predicted by a low Subjective Well-being under Neuroleptic Treatment scale (SWN-K) total score at baseline, initial treatment with conventional antipsychotics, and further by medication non-adherence as well as not being in early symptomatic and functional recovery at 3 months. Stable moderate cluster affiliation was predicted by being employed, a higher SWN-K total score at baseline, and further by non-adherence with medication. Early improvement cluster affiliation was mainly predicted by a good functional level at baseline, initial treatment with atypical antipsychotics and further by symptomatic recovery at 3 months.

Reference	Country	Study design	Follow-up	Intervention	Participants	Aims	Main findings
51. (Lencucha et al., 2008)	Canada	qualitative study	n/a	n/a	8 patients with schizophrenia	To explore how individuals living with schizophrenia form and maintain social relationships.	The findings suggest that environmental proximity, a routine environment, reciprocity, constancy/hope, and understanding are important dimensions in the formation and maintenance of social relationships.
52. (Li & Arthur, 2005)	China	randomized controlled trial	9 months	family education vs. standard care	101 patients with schizophrenia and their families	To examine the effect of patient and family education on families' knowledge about schizophrenia, and on patients' symptoms, psychosocial functioning, adherence to medication regimens and relapse rates.	The education program had a significant positive effect on families' knowledge and on patient symptoms and overall functioning. There were no significant differences between the experimental and control groups in medication adherence and relapse rates. Patients who were non-adherent to medication regimens were more likely to relapse.
53. (Lincoln & Hodgins, 2008)	Canada, Finland, Germany, Sweden	longitudinal observational study	2 years	n/a	216 patients with schizophrenia	To examine the contribution of low insight to the prediction of aggressive behavior among a high-risk sample of individuals with schizophrenia.	The prevalence of physically aggressive behavior in the examined sample was rather low. Lack of insight was associated with aggressive behavior in univariate analyses, but did nor contribute to the prediction of aggressive behavior, once scores for psychopathy and positive symptoms were entered into the model. Aggressive behavior was more strongly associated with high scores for psychopathy traits and positive symptoms than with lack of insight.
54. (Liu et al., 2007)	Canada	qualitative study	n/a	n/a	7 patients with schizophrenia	(1) To explore participants' experiences of a supported-employment program. (2) To gain insights into what processes and outcomes are meaningful and important for participants and may enable an evaluation of such	A tentative grounded theory was developed to with three themes of supported-employment program outcomes: (1) removing barriers to job seeking, (2) improving psychological well-being, and (3) participating in work. The program removed barriers to job seeking by (a) supporting effective

Reference	Country	Study design	Follow-up	Intervention	Participants	Aims	Main findings
						processes and outcomes for their congruence with occupational therapy practice.	job seeking, (b) improving work-related skills and knowledge, and (c) encouraging a partnership between case managers and participants. Two distinct phenomena related to sustained positive changes in participants' psychological well-being were identified: (a) becoming reassured and (b) achieving a better self-image. Participating in work was not perceived as a direct consequence of supported-employment outputs; rather it was a product of combining one outcome theme (removing barriers to job seeking) and two intervening conditions (participant's amount of effort in job seeking and readiness for job seeking).
55. (Magliano et al., 2006)	Italy	randomized controlled trial	6 months	family psychoeducation vs. control group assigned to a waiting list	71 patients with schizophrenia,126 relatives	To explore the effects of a psychoeducational family intervention for schizophrenia on patients' personal and social functioning as well as on relatives' burden and perceived support.	A significant improvement was found for the intervention group in patients' social relationships, interests in obtaining a job, maintenance of social interests, and management of social conflicts. For both the intervention and control groups, family burden significantly improved. Relatives' social contacts and perception of professional support significantly increased only in the intervention group.
56. (McCreadie et al., 2005)	Scotland	randomized controlled trial	18 months (6-month interventio, 12-month post-intervention follow-up)	provision of free fruit and vegetables with associated instruction in meal planning and food preparation vs. provision of free fruit and vegetables without instruction	102 patients with schizophrenia	To measure the impact of giving free fruit and vegetables for 6 months on eating habits in schizophrenia.	The diet of participants improved when they were given free fruit and vegetables but this was not sustained after withdrawal of the intervention. A support program added no benefit. There was no between-group difference at any time point in measures of change from baseline in psychopathological symptoms, blood indices of nutrition,

Reference	Country	Study design	Follow-up	Intervention	Participants	Aims	Main findings
				vs. treatment as usual			body mass index, level of physical activity and risk of coronary heart disease.
57. (Meijer et al., 2009)	Netherlands	longitudinal observational study	18 months	n/a	143 patients with schizophrenia	To examine the mediating role of health related quality of life (HRQoL) in the prediction of general quality of life (GQoL) and determining the temporal stability of these findings.	GQoL was predicted mainly by anxiety and depression and self-esteem and to a lesser extent by global functioning and social integration. At both time intervals HRQoL appeared to be a significant mediator of the relationship between anxiety and depression and self esteem versus GQoL.
58. (Naber et al., 2005)	Germany	randomized controlled trial	26 weeks	olanzapine vs. clozapine	114 patients with schizophrenia	To evaluate the effects of olanzapine versus clozapine on subjective well-being, quality of life (QOL) and clinical outcome.	Both atypical antipsychotics were found to improve patients subjective well-being, as measured by the Subjective Well-being under Neuroleptic Treatment (SWN) scale. Olanzapine was non-inferior to clozapine regarding the improvement in SWN total scores. Quality of life, as assessed by the Munich Life Dimension List (MLDL), also significantly improved under treatment with both antipsychotics. Furthermore, psychopathology was reduced similarly with both compounds. The effects of antipsychotic treatment on subjective well-being (SWN) correlated only moderately with their effects on psychopathology (PANSS). The proportion of patients who experienced treatment-related adverse events was lower in the olanzapine than in the clozapine group, and physicians' side effect ratings were better in the olanzapine group.
59. (Nathans-Barel et al., 2005)	Israel	controlled clinical trial	10 weeks	dog-assisted therapy vs. comparable	20 patients with schizophrenia	To study the effectiveness of animal-assisted therapy (AAT) in the treatment of	The AAT group showed a significant improvement in the hedonic tone compared to controls. The experimental

Reference	Country	Study design	Follow-up	Intervention	Participants	Aims	Main findings
				treatment sessions without a dog		anhedonia in schizophrenia patients.	participants also showed an improvement in the use of leisure time and a trend towards improvement in motivation. No significant differences between the two groups were found in psychiatric symptoms.
60. (Nolan et al., 2009)	USA	longitudinal observational study	8-12 weeks	The data were collected in a randomized clinical trial of the anti-aggressive efficacy of adjunctive valproate.	42 patients with schizophrenia	To describe the reasons for aggressive behavior reported by patients and staff and the relationships between those reasons and the subsequent interventions delivered in response to aggression.	There were 317 aggressive incidents. Verbal aggression was most frequent (244 incidents), followed by physical aggression against other people (100 incidents) and aggression against objects (31 incidents). Patients reported more often than staff that aggression was provoked by external factors (e.g., interpersonal conflict). Staff cited internal factors (e.g., psychotic symptoms) more often than patients. Interventions delivered to control aggression were related to the type and severity of aggression, rather than to the reasons for aggression.
61. (Novick et al., 2009)	10 European countries	longitudinal observational study	3 years	n/a	6642 patients with schizophrenia	To determine the frequency and predictors of recovery among outpatients with schizophrenia after 3 years of treatment.	Of the 6642 patients analyzed, 33% achieved long-lasting symptomatic remission, 13% long-lasting functional remission, 27% long-lasting adequate quality of life, and 4% achieved recovery during the follow-up period. Social functioning at study entry was one of the most important predictors of recovery. Patients were less likely to achieve recovery if they had more severe negative symptoms, higher BMI at baseline and lack of effectiveness as the reason for change of medication at baseline. Adherent patients were more likely to achieve recovery than non-adherent patients. Taking olanzapine was associated with a higher likelihood

Reference	Country	Study design	Follow-up	Intervention	Participants	Aims	Main findings
							of recovery compared with taking risperidone, quetiapine, two or more antipsychotic medications or typical antipsychotics.
62. (Olié et al., 2006)	international study ("26 centers in Western Europe")	randomized controlled trial	12 weeks	ziprasidone vs. amisulpride	123 patients with schizophrenia	(1) To compare the efficacy of ziprasidone and amisulpride in the treatment of negative symptoms in schizophrenic subjects with predominantly negative symptomatology. (2) To compare the efficacy of ziprasidone and amisulpride in the treatment of general psychopathology and global illness severity. (3) To evaluate the safety and tolerability of the two agents.	PANSS Negative Subscale score, the primary efficacy variable, improved for both treatment groups over the study period. Ziprasidone demonstrated efficacy comparable to amisulpride in improving negative symptoms, global psychopathology, and global functioning. Both agents were generally well tolerated, with comparably low incidences of movement disorders.
63. (Penadés et al., 2006)	Spain	randomized controlled trial	10 months (4-month intervention, 6-month post-intervention follow-up)	Cognitive Remediation Therapy (CRT) vs. Cognitive Behavioral Therapy (CBT)	40 patients were randomized to either CRT or CBT and 20 patients receiving standard treatment were used to establish reliable change.	To examine the effects of Cognitive Remediation Therapy (CRT) on neurocognition, and secondarily on symptomatology and psychosocial functioning.	CRT produced an overall improvement in neurocognition, particularly in verbal and nonverbal memory, and executive function. Furthermore, patients receiving CRT showed improvement in social functioning, specifically in communication and personal autonomy. CBT showed only a slight non-specific improvement in neurocognition (working memory). The group that received the CRT improved significantly over time on the PANSS cognitive factor, while the group that received the CBT showed a significant improvement on the depression/anxiety component of the PANSS.
64. (Puschner et al., 2009)	Netherlands, Germany, England,	longitudinal observational study	1 year	The data were obtained from a randomized	373 patients with schizophrenia	To examine the relationship over time of adherence to antipsychotic medication	For most variables included in the model, a positive change in time was observed: increase in adherence,

Reference	Country	Study design	Follow-up	Intervention	Participants	Aims	Main findings
	Italy			controlled trial evaluating the effects of adherence therapy in comparison to health education.		and quality of life (QoL) in people with schizophrenia, taking into account effect of mediating variables.	insight, QoL, and level of functioning, and decrease in symptom severity. Only attitude towards medication and rating of severity of unwanted side-effects remained stable. Some links of either adherence or QoL with other variables found at baseline could be confirmed at follow-up (side effects – QoL, symptoms – QoL, attitude – QoL), but this did not apply to the one link identified for adherence at baseline (adherence – symptoms). Including time course in the analysis suggests that the pathway by which adherence might affect QoL across time is via baseline symptoms, side effects and/or QoL at baseline. The findings did not support direct links between adherence and QoL.
65. (Ramírez García et al., 2006)	USA	longitudinal observational study	9 months	n/a	30 patients with schizophrenia, 30 family caregivers	To test whether family support predicts medication usage among Mexican American individuals with schizophrenia.	Of the 30 schizophrenia patients, 13 (43%) were classified as regular medication users and 17 (57%) as irregular. Family instrumental support predicted higher medication usage (Odds Ratio=4.8) in multivariate analyses that statistically adjusted for the impact of emotional support, family expressed emotions, and psychiatric status on medication usage. Emotional support and expressed emotions were not significant predictors of medication usage. The data also suggest that warmth (positive affect) is an unlikely mediator between instrumental support and medication usage.
66. (Resnick et al., 2008)	USA	randomized controlled trial	18 months	olanzapine vs. quetiapine vs. risperidone vs.	1121 patients with schizophrenia	To examine the relationship between assignment to five different antipsychotic	Rates of employment varied considerably throughout the trial, with a low of approximately 15% at baseline

Reference	Country	Study design	Follow-up	Intervention	Participants	Aims	Main findings
				ziprasidone vs. perphenazine		medications and two outcomes: employment and participation in psychosocial rehabilitation (PSR).	for those assigned to perphenazine and risperidone, to a high of slightly over 30% for those on ziprasidone and perphenazine at 18 months. There was a moderate but significant relationship between employment status and participation in PSR, suggesting that participation in PSR is associated weakly but significantly with current employment. There were no differences between medication groups on employment outcomes or participation in PSR.
67. (Rice, 2008)	USA	qualitative study	n/a	n/a	9 women with schizophrenia, 11 case managers	(1) To examine violence perpetrated against women diagnosed with schizophrenia. (2) To understand how this population of women has become "invisible" and an acceptable victim of violence. (3) To critically examine the perspectives of women who experience violence and case managers who care for them.	Stigma was an overarching theme in the stories of women and case managers. Women believed they were acceptable victims of violence. They knew that the coexistence of violence and schizophrenia contributed to their stories of violence being doubted or disbelieved. Providers substantiated women's understanding that no one would listen to their stories. Women also believed that schizophrenia was the "lowest" on a hierarchy of mental illnesses and providers substantiated this belief. Providers and consumers accepted violence among women as "the way things are." Neither group expressed hope for possibilities of change that violence could be overcome. Although providers were aware that stigma existed for women, they perceived that stigma was found elsewhere, not within their own relationships with women.
68. (Riedel et al., 2005)	Germany	randomized controlled trial	12 weeks	quetiapine vs. risperidone	44 patients with schizophrenia	To compare the efficacy and safety of risperidone and	Both antipsychotics produced significant decreases in overall,

Reference	Country	Study design	Follow-up	Intervention	Participants	Aims	Main findings
						quetiapine in patients with schizophrenia presenting with predominantly negative symptoms.	positive and negative symptoms. Quetiapine and risperidone proved to be similarly efficacious in all clinical measures over the 12-week period, with no significant difference in PANSS total scores, PANSS positive and negative subscores, CGI scores and SANS scores. The tolerability profile of quetiapine was advantageous compared with risperidone.
69. (Ritchie et al., 2006)	Australia	randomized controlled trial	6 months	olanzapine vs. risperidone	66 patients with schizophrenia	To determine whether either olanzapine or risperidone was superior in terms of efficacy or side effects when treating schizophrenia in late life.	Parkinsonism, positive and negative symptoms of schizophrenia improved in both groups both from baseline switch to olanzapine or risperidone and during the six-month follow-up. There was no difference at six-month follow-up between the groups with regard to depressive symptoms, though the olanzapine group exhibited a significant decline in depressive symptoms from baseline to the end of the six-month comparison. No such change was experienced by the risperidone group. Furthermore, patients treated with olanzapine showed a significantly greater improvement in quality of life.
70. (Ritsner et al., 2006)	Israel	longitudinal observational study	16 months	n/a	148 patients with schizophrenia	To identify factors that influence changes in satisfaction with quality of life (QoL) of schizophrenia patients.	Baseline levels of activation symptoms, emotional distress, task oriented coping, self-esteem and friend support accounted for 41% of the variability in the general QOL index at follow-up. Determinants of change in QoL over time were different being in hospital or out of hospital in the real world. A reduction in paranoid factor, and emotional distress together with increasing self-esteem and other

Reference	Country	Study design	Follow-up	Intervention	Participants	Aims	Main findings
							support ratings were associated with improvement in the general QoL index among patients who were reassessed during the same hospitalization. A decrease in anergia factor and emotional distress contributed to a better perception of QoL among patients who were reassessed at discharge from an additional admission. In the outpatient group, four negative (anergia, distress, side effects, and emotional related coping) and two positive (avoidance coping, expressed emotion) predictors of improvement of QoL were identified.
71. (Ritsner & Ratner, 2006)	Israel	longitudinal observational study	16 months	n/a	148 patients with schizophrenia	To define the long-term changes in coping strategies used by schizophrenia patients and their relation to clinical and psychosocial factors.	Four temporal coping types were distinguished: (1) stable favorable, (2) stable unfavorable, (3) becoming favorable, and (4) becoming unfavorable. Either favorable or unfavorable coping patterns of 62.2% patients remained stable over time, and they changed to unfavorable among 19.6% of the patients and to favorable among 18.2% of the patients. Each temporal coping type was associated with a specific pattern of changes in clinical and psychosocial variables. Particularly, the becoming favorable coping type was accompanied by a significant reduction of all symptom intensity, and improvement in psychosocial variables. The stable favorable coping type showed decreasing severity of dysphoric mood and emotional distress with elevation of self-esteem and social support scores over time. The stable unfavorable

Reference	Country	Study design	Follow-up	Intervention	Participants	Aims	Main findings
							coping type was associated with stable over time scores of clinical and psychosocial variables. The becoming unfavorable coping type related to worsening autistic preoccupation and self-efficacy.
72. (Ritsner et al., 2007)	Israel	longitudinal observational study	16 months	n/a	148 patients with schizophrenia, 175 healthy controls (hospital staff members)	To test the hypothesis that a positive family history of schizophrenia and related psychoses is associated with a higher level of emotional distress in schizophrenia subjects.	There were 79 patients with a negative family history and 69 patients with a positive family history. No between-group differences were found in psychopathological symptoms and side effects of medication. Both groups had similar levels of emotional distress at baseline. Patients with a negative family history reported improvements in distress and depression severity after 16 months, while those with a positive family history experienced persistent elevated emotional distress, mainly, on obsessiveness, and depression subscales. Both groups of patients were characterized by elevated emotional distress at follow-up examination compared to healthy subjects.
73. (Russell et al., 2006)	England, Australia	pilot intervention study	Training and assessments can be completed in a single session.	micro-expressions training tool (METT) – a computer-based emotion recognition training package	20 patients with schizophrenia, 20 healthy controls	To investigate the effectiveness of a computer-based training package to help improve emotion recognition impairments in patients with schizophrenia.	Both patients with schizophrenia and healthy subjects made significant improvements in emotion recognition following the METT training. Patients with schizophrenia improved to a level that did not distinguish them from pre-trained controls. The change in performance was similar across groups, although the patient group required more METT training trials to make similar improvements.
74. (Ryu et al., 2006)	Japan	longitudinal observational study	2 years	n/a	78 patients with schizophrenia	To investigate the effects of deinstitutionalization and evidence-based strategies	In 2 years after hospital discharge, 60 (76.9%) patients remained in the community residence without having

Reference	Country	Study design	Follow-up	Intervention	Participants	Aims	Main findings
						for the treatment of mental disorders among long-stay patients after their discharge from a mental hospital and to assess the patients' social and clinical outcomes over 2-year post-discharge period.	experienced any interruptions from major exacerbations of their mental disorders or physical illness. Four (5.1%) patients had major psychotic or affective exacerbations and were readmitted to psychiatric wards. Twelve (15.4%) people were admitted to hospital because of serious physical illnesses. One resident committed suicide. Those who remained in the community facility for 2 years demonstrated significant improvements in the areas of psychiatric symptoms, global functioning, social activity, speech functions, self-care, general behavior, withdrawal, independence (performance), independence (competence), and employment. Unexpectedly, a deterioration on the measure of insight was observed.
75. (Salokangas et al., 2007)	Finland	longitudinal retrospective study	3 years	n/a	2502 patients with schizophrenia (three samples of patients, who were discharged from psychiatric hospitals in 1986, 1990, and 1994)	To study social role behavior (SRB) among patients with long-term schizophrenia living in the community during sharp decline in number of psychiatric beds.	About 85% of all patients had considerable problems in at least one of the measured areas of behavior, while one third had problems in five or more areas of behavior. The most problematic areas were work, initiative, close relationships, social withdrawal, social relationships and managing money. In the majority of SRB areas, men managed more poorly than women. Later discharge years were associated with poor SRB at follow-up. Being married and having good psychosocial functioning and ability to work, as well as having paranoid type of schizophrenia related to fewer problems in SRB at follow-up.

Reference	Country	Study design	Follow-up	Intervention	Participants	Aims	Main findings
76. (Salokangas et al., 2006)	Finland	longitudinal retrospective study	3 years	n/a	2221 patients with schizophrenia (three samples of patients, who were discharged from psychiatric hospitals in 1986, 1990, and 1994)	To explore the levels of life satisfaction among patients with long-term schizophrenia who were discharged from hospitals into the community and the factors associated with their satisfaction.	Patients discharged in 1994 were more satisfied than those discharged earlier. Patients tended to report more satisfaction at follow-up if they were female, had good psychosocial functioning, had several confidants, or were living in group homes or dormitories. Patients tended to be less satisfied at follow-up if they were divorced or had mental and physical symptoms. Patients with depressive symptoms or with low psychosocial functioning and those who were currently hospitalized reported the smallest changes in levels of satisfaction between the two time points. Patients with low psychosocial functioning who were living with their parents, in group homes or dormitories, or in institutions were relatively satisfied, whereas patients with good psychosocial functioning were more satisfied if they lived with their spouse or partner.
77. (Sánchez et al., 2009)	Spain	longitudinal observational study	6 months	n/a	95 patients with schizophrenia, 53 healthy controls	(1) To identify variables that predict functional disability in chronic schizophrenia over time. (2) To analyze the role played by verbal memory, working memory, and executive functioning in specific disability domains such as self-care, family contact, vocational outcome, and social functioning. (3) To explore the possible interaction between processing speed and other	Patients performed worse in all cognitive domains compared to healthy controls. The most impaired domain in the patient group was processing speed, followed by verbal memory. The level of disability in the sample was severe. The most impaired area was social functioning, followed by vocational outcome and family contact. Most cognitive measures were significantly related to the functional outcome domains 6 months after admission to the study. Results obtained support the hypothesis that processing speed acts as

Reference	Country	Study design	Follow-up	Intervention	Participants	Aims	Main findings
						cognitive domains as predictors of specific functional abilities observed in schizophrenia.	a pathway through which verbal memory, executive functioning, and working memory predict the course of patients' functional ability over time. Processing speed was found to be the best longitudinal predictor of the level of autonomy in patients with chronic schizophrenia.
78. (Schindler, 2005)	USA	controlled clinical trial with both quantitative and qualitative data collected	12 weeks	Role Development Program (RDP) vs. multi-departmental activity program (MAP)	84 patients with schizophrenia	To examine if adults diagnosed with schizophrenia demonstrated improved task and interpersonal skills and social roles when involved in an individualized intervention based on the Role Development Program (RDP), in comparison to an intervention based on a multi-departmental activity program (MAP).	There was a statistically significant improvement in task skills, interpersonal skills, and roles among participants involved in the experimental group (RDP). Qualitative findings supported the quantitative findings. Participants from both groups indicated that the rehabilitation program was a positive aspect of their hospitalization. However, for the comparison group this was primarily a productive use of time while the participants in the experimental group could cite specific skills and roles they learned through participation in the program. Also, only the participants in the experimental group could verbally identify long-term benefits and specific ways in which their roles and skills could transfer to other life situations.
79. (Schomerus et al., 2007)	UK, France, Germany	longitudinal observational study	2 years	n/a	1208 patients with schizophrenia	To determine the effect of rural or urban residence on frequency of social and family contacts.	Family visits, social visits and planned social activities were less common in urban compared to rural settings, whereas no significant differences were found for social and family telephone calls and time spent with a spouse. Women talked more frequently on the phone, both to friends and to family. Patients living with a partner had more contacts to family, but less social visits

Reference	Country	Study design	Follow-up	Intervention	Participants	Aims	Main findings
							and activities. Being widowed or separated increased phone calls with family members. Family and social contacts both declined with age across all categories. Employment increased social contacts, but did not influence contacts with other family members. Schizophrenia symptoms, particularly negative symptoms, and impaired global functioning decreased all aspects of social and family contacts. The country of residence was associated with meaningful differences in social, but not in family contacts.
80. (Schomerus et al., 2008)	UK, France, Germany	longitudinal observational study	2 years	n/a	1208 patients with schizophrenia	To determine how the experience of crime and subjective feelings of safety differ between patients with schizophrenia living in urban and rural areas.	One in 10 respondents reported at least one violent assault during the study period, and one in five at least one example of non-violent victimhood. There was no significant relationship between victim status and residential area. In contrast to objective criminal victimhood, the subjective sense of safety was clearly impaired in the urban environments. Being the victim of violence was most strongly associated with alcohol and drug abuse and with criminal arrests of the patients themselves, whereas impaired subjective safety was most strongly related to poverty and victimhood experience.
81. (Seo et al., 2007)	South Korea	controlled clinical trial	8 weeks	social skills training vs. routine nursing care	66 patients with schizophrenia	To examine the effects of social skills training on the social skills and self-esteem of inpatients with chronic schizophrenia.	The conversational, interpersonal relationship, and assertiveness skills, and self-esteem of the experimental group showed significant improvement, whereas problem-solving skills did not improve.

Reference	Country	Study design	Follow-up	Intervention	Participants	Aims	Main findings
82. (Siegel et al., 2006)	USA	longitudinal observational study	2-8 years (average 3 years)	n/a	98 patients with schizophrenia (45 first-episode patients and 53 previously treated patients)	To assess the relationship between symptoms and cognitive measures at intake and functional outcome 2-8 years later in first-episode and previously treated schizophrenia patients.	Patients' level of function increased over time; this change was also reflected in decreased levels of depressive, negative, and positive symptoms. The level of function at intake proved to be an important predictor of later functional outcome. Several symptom-related and demographic variables were also good predictors of future level of function. Specifically, a better functioning at follow-up was predicted by lower levels of positive, negative and depressive symptoms at intake. Symptoms at intake had distinct patterns of prognostic significance for functional outcome in previously treated patients, compared with first-episode patients. In addition, male and female patients differed in the degree to which initial symptoms were correlated with later function. Cognitive function at intake was not significantly associated with functional outcome at follow-up in the final multivariate model.
83. (Sim et al., 2006)	Singapore	longitudinal observational study	2 years	n/a	142 patients with schizophrenia	(1) To examine the prevalence of reported physical comorbidity in a cohort of patients with first episode schizophrenia. (2) To evaluate the longitudinal outcomes of these individuals with respect to the clinical and functional domains.	Physical comorbidity was present in 21.8% (n=31) of the patients: 16.9% (n=24) had co-existing medical illnesses, 4.2% (n=6) had co-existing surgical illnesses and 0.7% (n=1) had both at baseline. Compared to baseline measurements, patients with physical comorbidity had greater awareness into the consequences of their psychiatric illness at 12 month, the need for treatment at 12 and 18 months, and better improvement of PANSS total

Reference	Country	Study design	Follow-up	Intervention	Participants	Aims	Main findings
							and general psychopathology subscale scores at 24 months. Patients with physical comorbidity also had less reduction in their WHOQOL-Bref scores in the physical health domain at 12 and 18 months and greater increase in the GAF scores at 18 and 24 months, indicating better subjective rating of quality of life and objective measure of their global psychosocial functioning prospectively.
84. (Sirota et al., 2006)	Israel	randomized controlled trial	12 weeks	quetiapine vs. olanzapine	40 patients with schizophrenia	To compare the efficacy, safety and tolerability of the atypical antipsychotics quetiapine and olanzapine in treating the negative symptoms of schizophrenia.	Quetiapine and olanzapine were similarly effective: in each treatment group significant improvements at week 12 were observed for negative symptom scores on the SANS and the PANSS, and for subscale scores of affective flattening and alogia on the SANS. It was also shown that these two atypical antipsychotics produced a beneficial effect on other symptom domains, as assessed by the PANSS total and subscale scores. There were no significant differences in PANSS total and subscale scores between the two treatment groups at any assessment throughout the 12-week study. Both treatments were well tolerated, with no worsening of extrapyramidal symptoms in either case. Anxiety and insomnia were the two most commonly reported adverse events in both treatment groups.
85. (Soyka et al., 2007)	Germany	longitudinal retrospective study	7-12 years	n/a	1662 patients with schizophrenia	(1) To determine the prevalence of criminal acts post discharge among former inpatients with schizophrenia by reviewing	One hundred and sixty nine patients (10.17% of the total sample) committed criminal acts during the follow-up period. The register listed a total of 878 convictions for these patients. Of these

Reference	Country	Study design	Follow-up	Intervention	Participants	Aims	Main findings
						their earlier medical records, and the records in the German national crime register. (2) To determine predictors of later criminal behavior.	convictions, 94 were for violent crimes (physical aggression against other people), which were committed by 62 patients (3.73%). Five cases of manslaughter or murder were recorded (committed by 3 patients). Male gender was a substantial risk factor for non-violent and violent behavior. Significantly higher rates of criminal conviction and recidivism were found for patients with lack of insight at discharge. Analyses also showed a significantly higher risk of non-violent and violent crimes in patients with a hostility syndrome at admission and discharge. There was a significantly lower incidence of criminal behavior in subjects with a depressive syndrome.
86. (Srinivasa et al., 2005)	India	longitudinal observational study	18 months	n/a	100 patients with schizophrenia	To examine the costs associated with a community outreach program targeted at meeting the needs of people with schizophrenia living in rural India, and to assess its impact on the personal functioning of individuals with the disease itself as well as the associated burden on families.	Outreach management of persons with previously untreated schizophrenia resulted in significant reductions in positive and negative symptoms (by 58% and 43% respectively). A score for general psychopathology revealed less improvement. Symptomatic improvement was accompanied by considerable and sustained reductions in levels of disability (a 70% change on the WHODAS II summary score) and family burden (80%). The major improvements on these outcome measures were observed within the first 9 months. Increases in treatment and community outreach costs over the follow-up period were accompanied by reductions in the costs of informal-care sector visits and family care-giving time.

Reference	Country	Study design	Follow-up	Intervention	Participants	Aims	Main findings
87. (Strous et al., 2005)	Israel	longitudinal observational study	16 months	n/a	237 patients with schizophrenia	To examine task-, emotion-, and avoidance-oriented coping strategies in schizophrenia and explore associated clinical factors at exacerbation and stabilization phases of the illness.	Significant improvements over time were noted in psychopathological symptoms, emotional distress, insight, self-esteem, self-efficacy, perceived social support and quality of life. Patients tended to use more emotional coping strategies at exacerbation than at stabilization phase, whereas the task- and avoidance-oriented coping strategies remained unchanged in magnitude during the follow-up period. Regression analysis demonstrated emotional distress to be the strongest predictor of emotion-oriented coping, with self-efficacy and social support being the best predictors of task and avoidance coping strategies, respectively. Individual changes in these variables also appeared to be important predictors for fluctuations of these coping strategies over time.
88. (Swartz et al., 2007)	USA	randomized controlled trial	18 months	olanzapine vs. quetiapine vs. risperidone vs. ziprasidone vs. perphenazine	455 patients with schizophrenia	To compare the effects of olanzapine, quetiapine, risperidone, ziprasidone, and perphenazine, on psychosocial functioning in patients with chronic schizophrenia.	All antipsychotic treatment groups in all phases made modest improvements in psychosocial functioning. There were no differences among them after 6, 12, or 18 months. Significant positive predictors of improvement included lower baseline psychosocial functioning, female gender, abstinence from illicit substances, non-urban residence, higher baseline neurocognitive functioning, improvement in extrapyramidal symptoms, and improvement in CGI severity rating.
89. (Tabarés-Seisdedos et al., 2008)	Spain	longitudinal observational study	1 year	n/a	47 patients with schizophrenia, 43 patients with	To analyze if neurocognition and clinical status predicts the real-life	In schizophrenia patients, the variables at baseline that best predicted the functioning one year later were the

Reference	Country	Study design	Follow-up	Intervention	Participants	Aims	Main findings
					bipolar I disorder, 25 healthy control subjects	functioning for patients with schizophrenia or bipolar I disorder, using a longitudinal design.	composite neurocognitive score (CNCS), verbal memory and motor speed. The CNCS was the strongest predictor of good or low occupational adaptation group membership at follow-up. Symptoms appeared to explain less of the variance in functioning. Severity of general psychopathology was the only clinical significant predictor of subsequent functional outcome, and the depressive variable was the only clinical predictor of low occupational adaptation group membership. The variable that best predicted disability one year later was vocabulary domain. In bipolar I patients, changes in the CNCS over one year, deficits in the visual/motor processing domain, severity of symptoms and premorbid adjustment at the first assessment were the variables that better predicted functioning or disability changes over time.
90. (Temple & Ho, 2005)	USA	controlled clinical trial	6 months	Cognitive Behavior Therapy (CBT) vs. treatment-as-usual (TAU)	19 patients with schizophrenia	To determine the efficacy of Cognitive Behavior Therapy (CBT) in a population of medication-resistant patients with schizophrenia.	Statistically significant improvements were obtained in the CBT condition for the CGI-Improvement, Global Assessment Scale (GAS), and Global Psychosocial Functioning. CBT patients achieved a statistically significant decline in overall symptom severity, and in delusions. A trend toward reductions in negative symptoms was also achieved. Moderate to large effect sizes were achieved for overall life satisfaction, work functioning, and levels of global impairment, as well as for the CGI-Improvement, GAS, and Global

Reference	Country	Study design	Follow-up	Intervention	Participants	Aims	Main findings
							Psychosocial Functioning, and reductions in the severity of overall symptoms, delusions, and negative symptoms.
91. (Thirthalli et al., 2009)	India	longitudinal observational study	1 year	n/a	215 patients with schizophrenia	To examine the course of disability over a period of 1 year in three groups of community-dwelling schizophrenia patients: those who were already receiving antipsychotic treatment and continued to receive it, those who were not on treatment but started receiving antipsychotic medication during follow-up and those who were not on treatment and remained untreated.	Participants who were not receiving antipsychotics at baseline had significantly greater disability than those who were receiving antipsychotics. Those who continued to receive antipsychotics and those who started receiving them had a significant reduction in disability; this was apparent across all the four domains of disability (self-care, interpersonal relationships, communication and understanding, work) as well as in total disability. Disability remained virtually unchanged in those who remained untreated. Similar findings were observed for psychopathology scores. Across all the three groups, disability correlated highly with severity of psychopathology. Among the domains of psychopathology, the correlations were the strongest between the negative syndrome score and the total disability score.
92. (Usall et al., 2007)	10 European countries	longitudinal observational study	6 months	n/a	7990 patients with schizophrenia	To evaluate gender differences in schizophrenia in response to typical and atypical antipsychotics.	Women showed significantly greater improvement in overall clinical severity and quality of life compared with men. The analysis of the differences in response to treatment in positive, negative, depressive and cognitive symptoms has revealed that women respond better than men in all the different symptom dimensions. The highest gender differences were found in typical antipsychotics and clozapine.

Reference	Country	Study design	Follow-up	Intervention	Participants	Aims	Main findings
							Olanzapine only showed differences in quality of life, and no differences were found for risperidone.
93. (Valencia et al., 2007)	Mexico	randomized controlled trial	1 year	psychosocial skills training (PSST) + family therapy (FT) + treatment as usual (TAU) vs. treatment as usual (TAU) alone	82 patients with schizophrenia	To examine the effectiveness of a psychosocial skills training (PSST) approach applied to chronic outpatients with schizophrenia.	Patients in the intervention group improved their symptomatology, psychosocial and global functioning, showed lower relapse, rehospitalization and drop-out rates, a higher level of compliance with antipsychotic medication, and a high level of therapeutic adherence in comparison with patients receiving treatment as usual. Patients in the control treatment failed to improve in any of the clinical and psychosocial variables, their improvement was only in symptomatology. The effect sizes were large and medium for the intervention group in symptomatology, psychosocial functioning and global functioning, and small for the control group, with the exception of symptomatology, where the effect size was large for the two groups under study.
94. (Vauth et al., 2005)	Germany	randomized controlled trial	8-week intervention, 1-year post-intervention follow-up	computer-assisted cognitive strategy training (CAST) + vocational rehabilitation vs. training of self-management skills for negative symptoms (TSSN) + vocational rehabilitation vs. vocational rehabilitation alone (VRA)	138 patients with schizophrenia	To evaluate the effects of computer-assisted cognitive strategy training (CAST) and training of self-management skills for negative symptoms (TSSN) as adjuncts to vocational rehabilitation.	CAST appeared superior to VRA in measures of attention and verbal memory, and TSSN did not, pointing to a specific effect of the cognitive training. No significant difference was found for TSSN in improvements of negative symptoms. The rate of successful job placement in the 12-month follow-up was significantly higher for CAST than for VRA; neither TSSN and VRA nor CAST and TSSN differed significantly. CAST was 2.3 times as effective as VRA in returning people to work in the year following

Reference	Country	Study design	Follow-up	Intervention	Participants	Aims	Main findings
							termination of CAST. Improvement in short- and long-term verbal memory predicted a higher proportion of variance of successful job placement in the follow-up than pre-treatment history of employment alone.
95. (Villalta-Gil et al., 2009)	Spain	randomized controlled trial	12 weeks	Integrated Psychological Treatment (IPT) assisted by a dog vs. IPT alone	21 patients with schizophrenia	To assess the effectiveness of including a trained therapy dog in an intervention program applied to institutionalized patients with chronic schizophrenia.	Patients receiving an intervention assisted by a therapy dog showed significant improvements in the area of social contacts, in positive and negative and overall symptoms, and in quality of life related to social relationships. Patients receiving an intervention without a dog showed significant positive changes in positive, general and overall symptoms. No differences were found between the two groups before and after the application of the intervention.
96. Westheide et al., 2008)	Germany	longitudinal observational study	4 weeks	n/a	102 patients with schizophrenia	(1) To examine the relationship between sexual dysfunction, subjective well-being and prolactin levels in patients with schizophrenia treated either with risperidone or quetiapine. (2) To explore the relationship between testosterone and the severity of symptoms of schizophrenia in male patients.	After 4 weeks, patients treated with quetiapine reported less severe sexual impairment, as well as lower PANSS negative and general score compared with patients treated with risperidone. Additionally, emotional regulation as measured with the Subjective Well-being under Neuroleptic Treatment (SWN) scale was higher in patients treated with quetiapine. Risperidone was significantly associated with elevated prolactin levels. Prolactin levels were not correlated either with sexual dysfunction or PANSS. However, in the group of patients treated with risperidone, sexual impairment was significantly associated with the SWN subscale emotional regulation. No association

Reference	Country	Study design	Follow-up	Intervention	Participants	Aims	Main findings
							was found between PANSS negative score and testosterone in male patients as reported in other studies.
97. (Wölwer et al., 2005)	Germany	randomized controlled trial	6 weeks	training of affect recognition (TAR) vs. cognitive remediation training (CRT) vs. treatment as usual (TAU)	77 patients with schizophrenia	(1) To investigate the efficacy and specificity of a new training program particularly designed for the remediation of impairments in facial affect recognition. (2) To compare the effects of this "Training of Affect Recognition" (TAR) with a cognitive remediation program primarily aiming at improving attention, memory and executive functioning, and with treatment as usual without participation in a specific remediation program.	Patients receiving TAR significantly improved in facial affect recognition. Patients in the two control groups did not improve in facial affect recognition, though patients under CRT improved in verbal memory functions. Performance in facial affect recognition was positively associated with performance in measures of attention and comprehension of social scripts. Furthermore, improvement in facial affect recognition performance was positively related to clinical improvement regarding negative symptoms. However, these variables explained only a modest amount of variance in facial affect recognition.
98. (Wykes et al., 2005)	UK	randomized controlled trial	36 weeks	group cognitive behavior therapy (CBT) for voices vs. treatment as usual (TAU)	85 patients with schizophrenia	To test the effectiveness of group cognitive behavior therapy (CBT) on social functioning and severity of hallucinations in patients with schizophrenia.	Group CBT for voices was associated with benefits in social functioning with a modest effect size (0.63) six months after the end of therapy. There was no general effect of group CBT on the severity of hallucinations. However, there was a large cluster effect of therapy group on the severity of hallucinations such that they were reduced in some but not all of the therapy groups. Improvement in hallucinations was associated with having very experienced therapists and receiving therapy early in the trial. There was some evidence that people in the CBT group improved their self-esteem and acquired more coping strategies.

Reference	Country	Study design	Follow-up	Intervention	Participants	Aims	Main findings
99. (Xiang et al., 2010)	China	longitudinal observational study	1 year	n/a	116 patients with schizophrenia	To assess the changes in the quality of life (QoL) of schizophrenia patients and to identify their predictors over a 1-year follow-up.	During the follow-up period, patients experienced significant improvement in the majority of outcome measures, namely psychotic and depressive symptoms, extrapyramidal side effects, and the QoL domains of physical functioning, role limitations due to physical problems, social functioning, and role limitations due to emotional problems. However, some QoL domains including general health, vitality, and mental health did not significantly change and the social functioning and the bodily pain domain of QoL worsened by the end of the study. Social functioning was a predictor of baseline QoL and change at 1-year follow-up.
100. (Xiang et al., 2006)	China	randomized controlled trial	8-week intervention, 6-month post-intervention follow-up	Community Re-entry Module (CRM; a module of a standardized, structured social skills training program) vs. supportive counseling (SC)	96 patients with schizophrenia	To evaluate the efficacy of the Community Re-entry Module (CRM) in comparison with routine supportive counseling (SC) with regard to psychiatric symptoms, social functioning, rate of relapse, and re-hospitalization.	Participants in the CRM group fared significantly better than their counterparts with respect to negative symptoms at post-intervention and at 6-month follow-up, general psychopathology at post-intervention and at follow-up, and social functioning at post-intervention and at follow-up. In the SC group, the positive and negative symptoms markedly deteriorated by the 6-month follow-up. Having controlled the pre-intervention scores, there were still significant differences between CRM and SC groups in negative symptoms, general psychopathology and social functioning at post-treatment and at follow-up and positive symptoms at follow-up. Rates of relapse and re-hospitalization in the CRM group were lower, although the

Reference	Country	Study design	Follow-up	Intervention	Participants	Aims	Main findings
							difference between the two groups was not statistically significant.
101. (Xiang et al., 2007)	China	randomized controlled trial	4-week intervention, 2-year post-intervention follow-up	Community Re-entry Module (CRM; a module of a standardized, structured social skills training program) vs. group psychoeducation	103 patients with schizophrenia	To evaluate the effectiveness of the Community Re-entry Module (CRM) in comparison with group psychoeducation with respect to social functioning, psychiatric symptoms and rates of re-employment, relapse and readmission to hospital in patients with schizophrenia.	The CRM group significantly improved in terms of social functioning, insight and psychiatric symptoms compared with the psychoeducation group. The re-employment rate was significantly higher and relapse and re-hospitalization rates were significantly lower in the CRM group.
102. (Yilmaz et al., 2008)	Sweden	qualitative study	n/a	n/a	4 patients with schizophrenia	To describe how persons with schizophrenia interact with others while performing everyday activities in different contexts in terms of facilitating and hindering factors.	The findings identified complex and dynamic descriptions of interaction in activity performance of the participants, which could be divided into two themes: (a) facilitating social interaction in activity performance and (b) hindering social interaction in activity performance. The findings also showed that dichotomous contextual factors influenced the interactions. These factors were: (a) the type of activity being performed was experienced as meaningful/not meaningful to the participant, (b) the attitude was trusting/lacking trust, and (c) the location in which the activity and interaction took place, at home/outside the home.
103. (Yilmaz et al., 2009)	Sweden	qualitative study	n/a	n/a	4 patients with schizophrenia	To identify social processes of participation in performing activities of everyday life among persons with schizophrenia by looking at what	The findings of this study were presented in three common plots giving possible explanations to how social processes precede or aggravate participation in performing everyday activities. The first plot consisted of

Reference	Country	Study design	Follow-up	Intervention	Participants	Aims	Main findings
						characterizes the social processes that precede or aggravate participation.	significant events that were characterized in the analysis as *When I know that I am appreciated and not different from others I want to continue what I'm doing.* The second plot consisted of significant events characterized as *When I trust the people around me and have the possibility to mean something to them I do meaningful things.* Finally, the third plot consisted of significant events characterized as *When I know in advance what's going to happen I take part in discussions and mutual decision-making.*
104. (Zhong et al., 2006)	USA	randomized controlled trial	8 weeks	quetiapine vs. risperidone	673 patients with schizophrenia	To compare the efficacy and tolerability of quetiapine and risperidone in the treatment of schizophrenia.	Quetiapine and risperidone were similarly efficacious in treating acutely exacerbated patients with chronic schizophrenia. Improvements with both treatments were comparable on PANSS total, negative, and general psychopathology subscales. Risperidone-treated patients had a significantly greater improvement in PANSS positive subscale score among all patients, but not among completers. Improvements in PANSS response rates, Clinical Global Impression-Change (CGI-C), and cognitive and social function were similar between treatment groups. Somnolence was more common with quetiapine; extrapyramidal symptoms and elevated prolactin rates were significantly higher with risperidone.
