# Supplementary material for: Disability and schizophrenia: a systematic review of experienced psychosocial difficulties
Source: BMC Psychiatry. 2012 Nov 9;12:193. doi: 10.1186/1471-244X-12-193 (PMC3539983; doi:10.1186/1471-244X-12-193)
Supplement: Additional file 4 — Table S2 Variables associated with the intensity or course of the 10 most frequently studied PSD categories. [file 1471-244X-12-193-S4.pdf]

Additional File 4

Supplementary Table S2 Variables associated with the intensity or course of the 10 most frequently studied PSD categories

	PSYCHOSOCIAL DIFFICULTY									
**ASSOCIATED VARIABLES****	**Psychopathological symptoms**	**Disability/ Functioning***	**Emotional functions**	**Relationships with others**	**Quality of life/ Wellbeing***	**Cognitive functions**	**Employment**	**Energy and drive**	**Looking after ones' health**	**Health status***
**Accessibility**				+ (1)						
**Adherence to treatment**	+ (3)	+ (2)		+ (1)	+ (5)	+ (1)				
**Attitude towards treatment (positive)**	+ (1)	+ (1)			+ (1)	+ (1)			+ (1)	
**Body weight**	– (1)				– (1)					
**Cognitive functions**										
in general		+ (1)<br>+/0 (1)			– (1)	+/– (1)<br>–/0 (1)	+ (1)			
executive functions		+/0 (1)					+ (1)			
insight	+ (1)	+ (1)		+ (1)						
language		+/0 (1)								
memory		+ (1)<br>+/0 (1)		+ (1)			+ (2)			
**Communication difficulties**				– (1)						
**Comorbidities**										
physical comorbidity	–/0 (1)	+ (1)<br>– (1)			– (1)	+ (1)				+/0 (1)
substance abuse	– (1)	– (1)			– (1)	+ (1)				
**Demographics**										
age		– (2)		+/0 (1)<br>– (2)	– (1)					
gender (male)	– (2)	– (4)<br>–/0 (1)	– (1)	– (1)<br>–/0 (3)	– (1)	– (1)	– (2)	– (1)		– (1)
marital status (married)		+ (2)		+/– (1)	+/0 (1)		+ (1)			

ASSOCIATED VARIABLES**	PSYCHOSOCIAL DIFFICULTY									
	**Psychopathological symptoms**	**Disability/ Functioning***	**Emotional functions**	**Relationships with others**	**Quality of life/ Wellbeing***	**Cognitive functions**	**Employment**	**Energy and drive**	**Looking after ones' health**	**Health status***
educational level		+ (1)<br>+/0 (1)		+/– (1)		+/0 (1)	+/0 (1)			+/0 (1)
employment status (employed)	+ (2)	+ (6)		+/0 (1)	+ (4)<br>+/0 (1)		+ (4)			+ (1)
disability benefits							– (1)			
social economic status		+ (1)		+ (1)						+ (1)
urban residence		– (1)		–/0 (1)						
independent living	+ (1)	+ (3)			+ (1)					
residential placement	+/– (1)	–/0 (1)		–/0 (1)	–/0 (1)					–/0 (1)
living alone				–/0 (2)						–/0 (1)
country of residence (Germany vs. UK and France)		+/0 (1)		+/0 (2)						+/0 (1)
**Dependent behaviour**				– (1)						
**Disability/ Functioning**										
lesser disability/better global functioning	+ (3)	+ (2)	+ (1)	+ (1)<br>+/0 (1)	+ (3)	+ (1)	+/0 (1)		+ (1)	+ (1)
social functioning		+ (2)<br>– (1)		+/0 (1)	+ (2)					+ (1)
**Emotional problems**										
anxiety				– (1)	– (1)		– (1)			–/0 (1)
depression		– (3)		– (3)	– (2)		– (3)<br>–/0 (1)			– (1)<br>–/0 (2)
feelings of stress					– (1)					
flat affect		– (1)								
hostility				– (1)						
**Endocrine disturbances**									– (1)	

**ASSOCIATED VARIABLES****	**PSYCHOSOCIAL DIFFICULTY**									
	**Psychopathological symptoms**	**Disability/ Functioning***	**Emotional functions**	**Relationships with others**	**Quality of life/ Wellbeing***	**Cognitive functions**	**Employment**	**Energy and drive**	**Looking after ones' health**	**Health status***
**Energy and drive**										
motivation				+ (1)						
vitality					+ (1)					
**Family history of mental illness**			–/0 (1)							
**Health services use**										
duration of treatment					– (1)					
duration of hospitalizations		+/– (1)<br>– (1)					– (1)			
number of hospitalizations		– (1)								
**Hope**				+ (1)						
**Illness-related variables**										
duration of illness	– (1)	– (2)			– (2)					
age at illness onset		+ (1)								
age at first treatment		– (1)			– (1)					
age at first hospitalization		+ (1)								
age at discharge from hospital		– (1)								
duration of untreated psychosis		– (1)		– (1)	+/0 (1)		– (1)			–/0 (1)
stage of illness (earlier)	+ (1)	+ (1)								
disorganized or residual subtype of schizophrenia		– (2)								
**Location (outside the home)**				+ (1)						
**Obsessive symptoms**		– (1)								
**Participating in social activities**		+ (1)			+ (1)					

**ASSOCIATED VARIABLES****	**PSYCHOSOCIAL DIFFICULTY**									
	**Psychopathological symptoms**	**Disability/ Functioning***	**Emotional functions**	**Relationships with others**	**Quality of life/ Wellbeing***	**Cognitive functions**	**Employment**	**Energy and drive**	**Looking after ones' health**	**Health status***
**Patient treatment**										
medication	+ (18)<br>+/0 (4)	+ (7)<br>– (2)	+ (5)<br>+/0 (4)<br>+/– (2)	+ (3)<br>+/– (2)	+ (9)<br>+/0 (2)<br>+/– (2)	+ (6)<br>+/0 (2)	+ (3)	+ (3)<br>– (2)	+/– (2)	+ (2)<br>+/0 (2)<br>–/0 (1)
psychosocial treatment	+ (3)	+ (3)		+ (3)	+ (1)	+ (1)<br>+/– (1)	+ (4)		+/0 (1)	
psychological therapy: cognitive therapy	+/0 (1)	+ (1)				+ (1)<br>+/0 (3)				
psychological therapy: cognitive behavioural therapy	+/0 (2)	+ (2)	+ (1)			+/0 (3)	+/0 (1)			
day care				+ (1)						
community-based care	+ (1)	+ (1)								
alternative treatment	+ (1)<br>+/0 (2)	+ (2)	+ (1)<br>+/0 (1)	+/– (1)		+/0 (1)				
**Psychomotor disturbances**		– (1)<br>–/0 (1)		– (1)			– (2)			
**Psychopathological symptoms**										
global intensity of symptoms	– (3)	– (5)			– (3)	– (1)			– (1)	
negative symptoms	– (1)	– (1)		– (1)<br>–/0 (1)	– (3)	– (1)<br>–/0 (1)	+/0 (1)<br>– (1)			– (1)
positive symptoms	– (1)<br>–/0 (1)	– (2)<br>–/0 (1)	– (1)	– (3)<br>–/0 (1)	+ (1)<br>– (3)		– (1)<br>–/0 (1)		– (1)	–/0 (1)
general psychopathology		–/0 (1)		+/0 (1)						
**Psychopathy traits**				– (1)						
**Quality of life/ Wellbeing**										
quality of life	+ (1)	+ (1)							+ (1)	
wellbeing	+ (2)	– (1)			+ (3)				+ (1)	

**ASSOCIATED VARIABLES****	**PSYCHOSOCIAL DIFFICULTY**									
	**Psychopathological symptoms**	**Disability/ Functioning***	**Emotional functions**	**Relationships with others**	**Quality of life/ Wellbeing***	**Cognitive functions**	**Employment**	**Energy and drive**	**Looking after ones' health**	**Health status***
**Relationships with others (positive)**										
no history of aggressive behaviour				+ (1)						
in general		+ (2)		+/0 (1)	+ (1)					
family relationships				+/0 (1)						
relationships with health professionals					+ (1)					
**Self-esteem**					+ (2)					
**Sense of meaning**				+ (1)						
**Side effects of medications**	– (1)	– (3)			– (2)	+ (1)<br>– (1)			– (1)	
**Skills**							+ (1)			
**Social cognition**				+ (1)						
**Social support**										
in general				+ (2)	+ (1)<br>+/0 (1)					+/0 (1)
family support									+ (1)	
friend support					+ (1)					
**Support for caregivers**										
in general	+ (1)	+ (1)		+ (1)			+ (1)		+ (1)	
education	+ (2)	+ (5)		+/0 (1)						
mutual support		+ (1)								
**Task-oriented coping**					+ (1)					
**Transportation**							+ (1)			
**Trust in others**				+ (1)						
**Year of discharge (1980s vs. 1990s)**					– (1)		+ (1)			

* Global scores or global concepts closely related to PSDs.

** It can be noticed that on several occasions PSD categories and their related factors are the same. This occurred when: 1) PSDs and factors associated with them, although not identical, in the process of data extraction and analysis were classified under the same general categories; 2) PSDs at some point in time were predicted by their previous (e.g. baseline or premorbid) levels.

Type of association with PSDs:
+ denotes association with positive outcomes, i.e. with lesser intensity of PSDs or with PSDs improvement over time;
– denotes association with negative outcomes, i.e. with greater intensity of PSDs or with PSDs deterioration over time;
+/0 denotes partial association with positive outcomes;
–/0 denotes partial association with negative outcomes;
+/– denotes association with both positive and negative outcomes within the same psychosocial area.

An association was regarded as partial if: 1). was found only in a subgroup of participants; or 2). a variable was found to be associated with some, but not all aspects of a given PSD category (e.g. with “cognitive functions: attention”, but not with “cognition functions: memory”); or 3). a variable was associated with some, but not all measures of the same PSD; or 4). some, but not all associated variables, which in a single study were classified under the same category (e.g. various neuroleptics falling under one common category: “patient treatment: medication”) were found to be significantly related to a given PSD category.

Numbers in brackets denote numbers of studies in which a given type of association was found.
